# Receptor Elimination by E3 Ubiquitin Ligase Recruitment (REULR): A Targeted Protein Degradation Toolbox

**DOI:** 10.1021/acssynbio.2c00587

**Published:** 2023-04-03

**Authors:** Dirk H. Siepe, Lora K. Picton, K. Christopher Garcia

**Affiliations:** † Department of Molecular and Cellular Physiology, Stanford University School of Medicine, Stanford, California 94305, United States; ‡ Department of Structural Biology, Stanford University School of Medicine, Stanford, California 94305, United States; § Howard Hughes Medical Institute, Stanford University School of Medicine, Stanford, California 94305, United States

**Keywords:** targeted protein degradation, E3 ligase, receptor, induced proximity, REULR, fratricide, nanobody

## Abstract

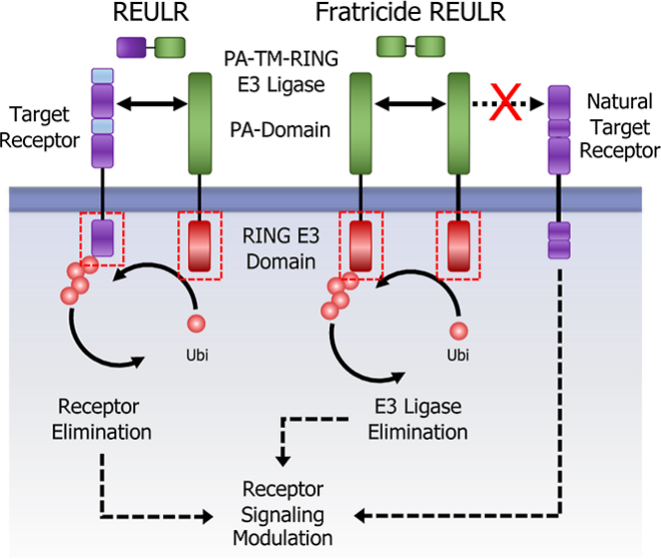

In recent years, targeted protein degradation (TPD) of plasma membrane proteins by hijacking the ubiquitin proteasome system (UPS) or the lysosomal pathway has emerged as a novel therapeutic avenue in drug development to address and inhibit canonically difficult targets. While TPD strategies have been successful in targeting cell surface receptors, these approaches are limited by the availability of suitable binders to generate heterobifunctional molecules. Here, we present the development of a nanobody (VHH)-based degradation toolbox termed REULR (Receptor Elimination by E3 Ubiquitin Ligase Recruitment). We generated human and mouse cross-reactive nanobodies against five transmembrane PA-TM-RING-type E3 ubiquitin ligases (RNF128, RNF130, RNF167, RNF43, and ZNRF3), covering a broad range and selectivity of tissue expression, with which we characterized the expression in human and mouse cell lines and immune cells (PBMCs). We demonstrate that heterobifunctional REULR molecules can enforce transmembrane E3 ligase interactions with a variety of disease-relevant target receptors (EGFR, EPOR, and PD-1) by induced proximity, resulting in effective membrane clearance of the target receptor at varying levels. In addition, we designed E3 ligase self-degrading molecules, “fratricide” REULRs (RNF128, RNF130, RENF167, RNF43, and ZNRF3), that allow downregulation of one or several E3 ligases from the cell surface and consequently modulate receptor signaling strength. REULR molecules represent a VHH-based modular and versatile “mix and match” targeting strategy for the facile modulation of cell surface proteins by induced proximity to transmembrane PA-TM-RING E3 ligases.

## Introduction

Classical drug discovery approaches against membrane protein targets such as cell surface receptors generally rely on small molecule inhibitors and monoclonal antibodies, but the vast majority of disease-relevant cell surface receptors still remain extremely challenging to target and have been largely deemed “undruggable” by established screening strategies.^1^ Finding alternative strategies to target challenging plasma membrane proteins has therefore become a prime focus in recent years.

Targeted protein degradation has emerged as a novel therapeutic strategy in drug development by directing proteins to the cells’ own degradation machinery (UPS).^2−4^ The majority of degraders such as PROTACs,^2^ molecular glues,^5^ dTags,^6^ or TRIM-Away^7^ are based on a heterobifunctional design that leads to the formation of a ternary complex between a cytosolic E3 ubiquitin ligase and a protein of interest to facilitate ubiquitination and subsequent 26S proteasome-dependent degradation.^8^ While classical degraders have been successful,^1^ this approach is ultimately limited to cytosolic targets, and therefore 1/3 of the protein-coding genes representing the membrane proteome are not accessible by this approach.^9,10^

More recently, targeted protein degradation approaches utilizing lysosomal degradation strategies (LYTAC and KineTac)^11,12^ and proteolysis-targeting antibodies (AbTac and ProTab) using WNT-related transmembrane E3 ligases (RNF43 and ZNRF3) have emerged.^13,14^ These approaches tether target proteins on the cell surface to either lysosome shuttling receptors or cell–surface E3 ubiquitin ligases to induce membrane clearance. Both technologies are mainly limited by the availability and specificity of shuttling receptors or transmembrane E3-binding moieties, selectivity (tissue expression), design (antibody formatting), and complexity of production.

In an effort to accelerate the development of targeted protein degradation tools, we present a modular and versatile nanobody (VHH)-based protein degradation toolbox termed REULR (Receptor Elimination by E3 Ubiquitin Ligase Recruitment). We generated human and mouse cross-reactive nanobodies against ECDs (extracellular domain) of five transmembrane PA-TM-RING-type E3 ubiquitin ligases (RNF128, RNF130, RNF167, RNF43, and ZNRF3), covering a broad range and selectivity of tissue expression. Next, we utilized our VHHs to characterize the expression of these five PA-TM-RING E3 ligases in commonly used human and mouse cell lines and immune cells (T cells, monocytes, B cells, and NK cells). We demonstrate that heterobifunctional REULR molecules can enforce transmembrane E3 ligase interactions with a variety of disease-relevant target receptors (EGFR, EPOR, and PD-1) by induced proximity, resulting in robust membrane attenuation of the target receptor. Furthermore, we present a strategic approach to tune transmembrane E3 ligases itself by generating homo-, heterobifunctional, and arrayed multimeric fratricide REULRs and consequently modulate signaling events of natural target receptors.

## Results

### PA-TM-RING E3 Ligase Nanobodies for Receptor Elimination

**Figure 1:**
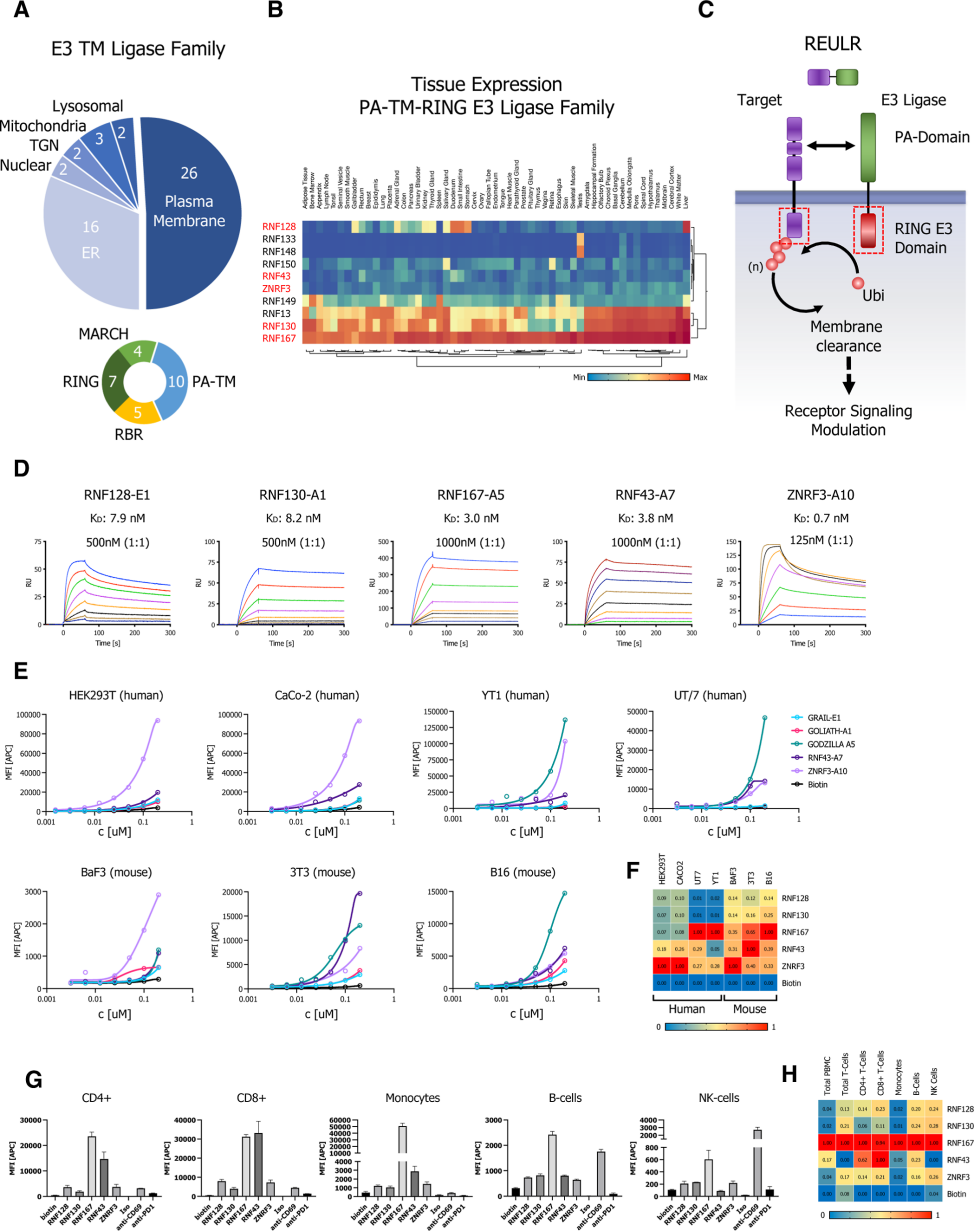
Transmembrane PA-TM-RING E3 ligase nanobodies for receptor elimination. (A) Pie chart representation of the transmembrane E3 ligase family classified by subcellular localization (upper chart). Plasma membrane-localized transmembrane E3 ligase subfamily grouped into subcellular and structurally related sub classes (lower chart). (B) Hierarchical two-way clustering heatmap of normal tissue mRNA expression data for the PA-TM-RING E3 ligase subfamily. (C) Schematic representation of the REULR concept. Enforced transmembrane E3 ubiquitin ligase recruitment to a target receptor reduces target receptor cell surface levels by E3 ligase-dependent intracellular ubiquitination and subsequent membrane clearance. (D) SPR sensograms and binding affinities of PA-TM-RING ligase-selected nanobodies (analytes) for human RNF128, RNF130, RNF167, RNF43, and ZNRF3 ECDs (ligands). (E) Cell surface staining of representative human (HEK293T, CaCo-2, YT1, and UT/7) and mouse (BaF3, 3T3, and B16) cell lines using a panel of five PA-TM-RING E3 ligase-binding nanobodies (nanobody:SA647 tetramers) and analysis by flow cytometry, full titration (1:1 dilutions; 200 nM tetramer), and Biotin:SA647 (Biotin) served as a negative control. (F) Staining data visualized in a normalized heatmap for human and mouse cell lines. (G) PBMC (Peripheral Blood Mononuclear Cells) immunophenotyping panel to identify the binding of five PA-TM-RING E3 ligase-binding nanobodies (nanobody:SA647 tetramers; 200 nM) to T cells (CD4+; CD8+), monocytes, B cells, and NK cells, analysis by flow cytometry. Biotin:SA647 served as a negative control (Biotin). Anti-PD1 and anti-CD69 were used as phenotyping control antibodies in comparison to an isotype control. (H) PBMC sub cell-type data summarized in a normalized heatmap. Data are represented as mean ± SD (*n* = 3).

The human transmembrane (TM) E3 ligase family represents a class of diverse RING-type E3 ubiquitin ligases^15,16^ with approximately 50 members (Figure 1A upper chart). These proteins exert widespread involvement in several diseases and cancer.^16,17^ The family can be further grouped into subcellular and structurally related sub classes; the plasma membrane localized E3 TM ligases include RING domain-containing proteins (7), PA-TM-RING (10), RING between RING (RBR; 5), and the membrane-associated RING-CH (MARCH; 4) families (Figure 1A, lower chart). In general, E3 ligases are notoriously challenging to study, and their substrates still remain highly elusive, mostly due to the nature of the ubiquitylation cascade, which is characterized by very weak target affinities and fast kinetics.^18−20^

Here, we focused on the PA-TM-RING-type E3 ligases,^21^ a family of approximately 10 members with a broad tissue expression pattern (Figure 1B) and a unique domain architecture that is minimally defined by three conserved domains: an extracellular protease-associated (PA) domain that acts as a substrate recruitment domain, a transmembrane domain (TM), and a cytosolic catalytic RING-type E3 ligase domain (RING-H2 finger; RNF) (Figure 1C). Mechanistically, the cytosolic RING E3 ligase domain functions as an allosteric activator and scaffold that recruits the ubiquitin machinery in close proximity to a substrate, while the extracellular PA domain functions as a substrate recruitment domain. We therefore hypothesized that PA-TM-RING E3 ligases could be retasked to selectively eliminate non-natural cell surface targets by an induced proximity approach that we termed REULR: Receptor Elimination by E3 Ubiquitin Ligase Recruitment (Figure 1C).

To develop a modular and versatile toolbox, we first identified five PA-TM-RING E3 ligases covering a wide range of tissue expression, allowing cell type-specific REULR approaches: RNF128 (GRAIL), RNF130 (GOLIATH), RNF167 (GODZILLA), RNF43, and ZNRF3 (Figure 1B; marked in red). Therapeutic monoclonal antibodies (mABs) and antibody engineering have revolutionized cancer therapies in the last decade,^22^ but they are not without limitations, mainly size, complexity of formatting, expression, and modularity. In order to overcome these limitations, we took advantage of the superior pharmacokinetic properties of nanobodies (VHH) such as their small size (1/10 the size of conventional antibodies), high stability, strong antigen-binding affinity, modularity, and ease of expression.^23−25^ We screened a synthetic nanobody library, allowing rapid high-throughput selection by yeast display^26^ using the ECDs (extracellular domains) of human RNF128 (GRAIL), RNF130 (GOLIATH), RNF167 (GODZILLA), RNF43, and ZNRF3 that led to 8 nanobodies against 5 ligases with nanomolar to picomolar affinities (Figures 1D and S1A–C).

A pairwise protein sequence alignment of the human and mouse ECDs of the five PA-TM-RING-type E3 ligases revealed that the ECDs are highly conserved between both species, with an average amino acid sequence identity of 97.75% (Figure S1D). We therefore tested our PA-TM-RING E3 ligase nanobodies against a panel of commonly used human (HEK293T, CaCo-2, YT1, and UT/7) and mouse (BaF3, 3T3, and B16) cell lines by cell surface staining as indicated (Figures 1E and S2A, B) and summarized in a normalized heatmap (Figure 1F). Indeed, all nanobodies tested were cross-reactive against human and mouse cell lines, which poses a significant advantage for the design and application of the REULR molecule for in vitro and in vivo studies. We next evaluated the nanobodies on primary cells using PBMCs (Primary Peripheral Blood Mononuclear Cells) to identify cell surface binding to immune cells: T cells (CD4+; CD8+), monocytes, B cells, and NK cells (Figures 1G and S2C), summarized in a normalized heatmap (Figure 1H). Similar to human and mouse cell lines, we observed some ligases like RNF167 being highly expressed in many cell types, while most other ligases tested show a more nuanced, cell type-specific expression pattern (Figure 1F,H).

### Receptor Elimination by E3 Ubiquitin Ligase Recruitment (REULR)

**Figure 2:**
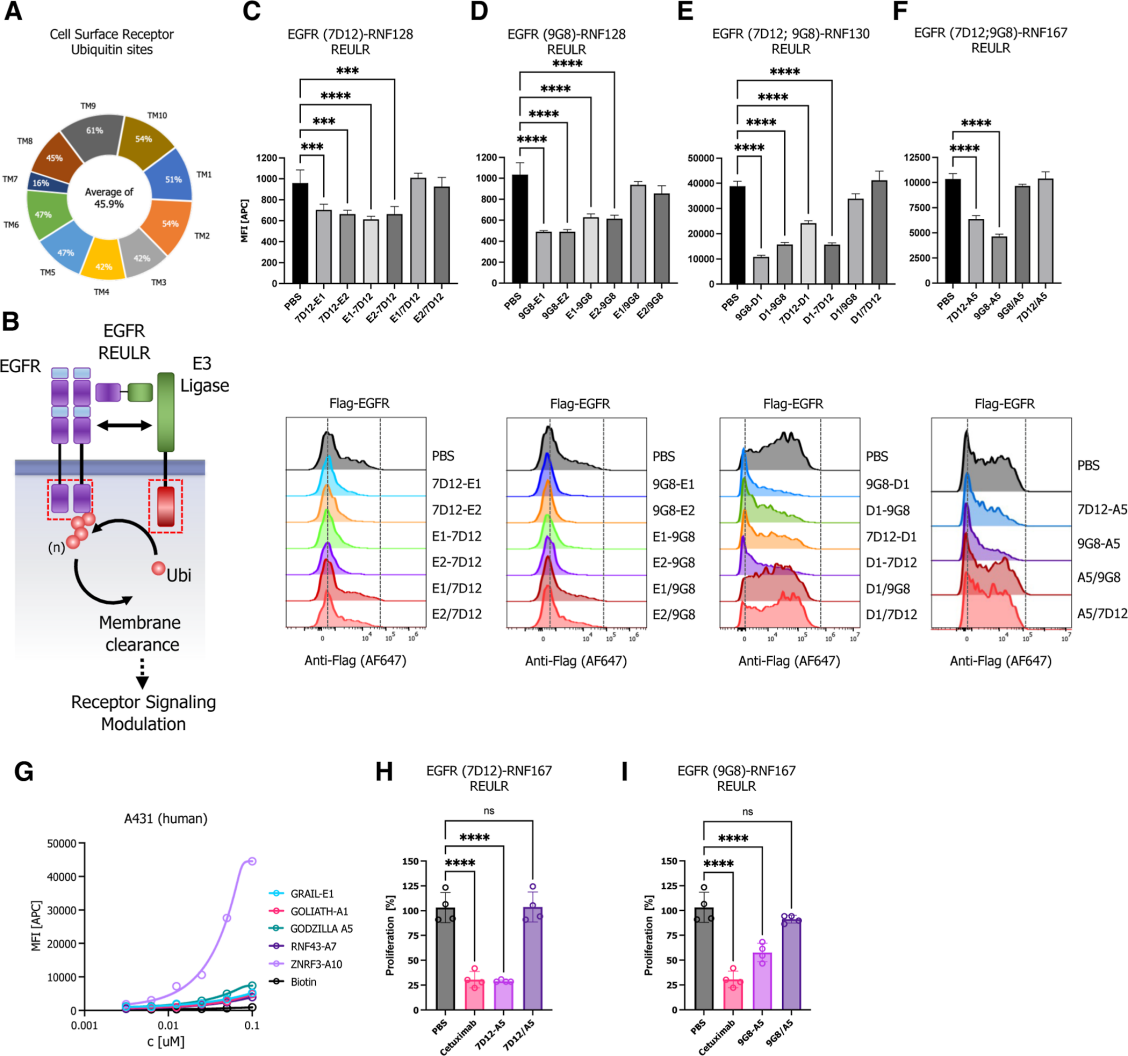
EGFR REULR. (A) Analysis of MS (Mass Spectrometry)-validated proteome wide ubiquitin sites matched to the human membrane proteome, subclassified by the number of transmembrane domains. (B) Schematic representation of EGFR degradation using a EGFR–REULR molecule. (C–F) HEK293T cells were transiently transfected with FLAG-tagged full-length EGFR cDNA (human) under the control of a constitutively active CMV (cytomegalovirus) promoter. 24 h post-transfection, cells were incubated with EGFR–REULR molecules (50 nM) as indicated using RNF128 (E1; E2)-, RNF130 (D1)-, or RNF167 (A5)-targeting nanobodies in combination with EGFR-binding moieties (7D12; 9G8) in varying orientations as indicated in comparison to monomeric nanobodies or PBS. After 24 h, cells were subjected to FACS analysis using a FLAG antibody (Alexa Fluor 647 conjugate) to monitor EGFR levels on the cell surface. Representative FACS histograms are visualized below the quantified data. Data are mean ± s.d. (*n* = 3 replicates). (G) Cell surface staining of A431 human squamous carcinoma cell using a panel of five PA-TM-RING E3 ligase-binding nanobodies (nanobody:SA647 tetramers) and analysis by flow cytometry, full titration (1:1 dilutions; 100 nM tetramer), and Biotin:SA647 (Biotin) served as a negative control. (H,I) Cell proliferation assay (CellTiter-Glo 2.0; Promega). A431 cells were seeded at 2.5k cells/well. After 24 h, cells were treated with PBS, cetuximab, or EGFR–REULR molecules as indicated using RNF167 (A5)-targeting nanobodies in combination with EGFR-binding moieties (7D12; 9G8) (50 nM). Cells were incubated for 72 h, washed, and subjected to CellTiter-Glo (2.0) assays to measure cell proliferation, according to the manufacturer’s specifications (Promega). Data are presented as a percentage of untreated cells (*n* = 4).

To evaluate potential targets for our REULR approach, we performed a membrane proteome wide analysis of reported ubiquitin sites.^27^ On average, 45% of cell surface receptors were reported to have at least one or more ubiquitin site (Figure 2A), which represents an untapped potential for cell surface receptors to be targeted using a REULR strategy. Members of the receptor tyrosine kinase (RTK) family including cytokine receptors EpoR (via JAK2 *V617F*) and members of the epidermal growth factor receptor (ErbB; HER) family represent the most common oncogenic drivers of malignant carcinomas.^28−30^ However, despite their immense clinical relevance, conventional drug discovery approaches have shown limited efficacy and problems of resistance.^31,32^ These limitations are mainly due to the nature of primary and emerging secondary escape mutations in the receptor (EGFR *T790M*) and acquired resistance, as well as pathway mutations, e.g., JAK2 *V617F* (EPOR/TPOR) that lead to constitutive over activation and dysregulation with detrimental outcomes for patients.^33−36^

We first designed different combinations of heterobifunctional REULRs to EGFR using two VHH (7D12; 9G8) that were previously described to inhibit ligand binding to EGFR: nanobody 7D12 sterically blocks ligand binding to EGFR, similar to cetuximab, and 9G8 acts by inhibiting high-affinity ligand binding and dimerization.^37−39^ To assess whether EGFR can be degraded by this set of REULR molecules, we overexpressed FLAG-tagged full-length EGFR in HEK293T cells that endogenously express PA-TM-RING E3 ligases at varying levels (Figure 1E) and treated cells with intact REULR molecules, monomeric versions, or PBS as negative controls (Figures 2C–F and S3A–D). We observed EGFR degradation after treatment with EGFR–REULR molecules at varying efficiencies, depending on the choice of the E3-targeting ligase, EGFR VHH, and orientation (Figure 2C–F). Collectively, EGFR–REULR designs using the N-terminal 9G8 nanobody in combination with C-terminal RNF128, RNF130, or RNF167-targeting nanobodies performed better and resulted in more effective EGFR degradation compared to other designs.

Targeting the EGFR pathway with tyrosine kinase inhibitors (TKI; e.g., afatinib, erlotinib, gefitinib, and osimertinib) or monoclonal antibodies (e.g., cetuximab, panitumumab, nimotuzumab, and necitumumab) is a well-characterized strategy for treating cancers including lung adenocarcinomas (NSCLC) and squamous cell carcinoma (SCC), which are one of the most prevalent types of skin cancer.^33,40^ A431 cells, a human squamous carcinoma cell line, have amplifications of the EGFR gene and, as a consequence, express a high level of EGFR. In addition, A431 cells have been widely used for studying skin cancer as well as for pharmaceutical and biomedical purposes in vitro and in xenograft models.^41−43^ We therefore explored how EGFR REULR molecules can exert antiproliferative activity in A431 cells in comparison to cetuximab, a first-generation anti-EGFR chimeric antibody used for the treatment of metastatic colorectal cancer and head and neck cancers.^44,45^ We first evaluated the positive binding of our PA-TM-RING E3 ligase nanobodies in A431 cells by cell surface tetramer staining (Figure 2G) and selected RNF167 (A5)-based REULR molecules that were tested in HEK293 cells (Figure 2F) for the proliferation assays in A431 cells. Indeed, in agreement with our degradation assays, treatment of A431 cells with intact REULR molecules using nanobodies against RNF167 (A5) in combination with two EGFR nanobodies (7D12 and 9G8) resulted in a significant reduction of cell proliferation but with different efficacies compared to cetuximab (Figure 2H,I), while monomeric nanobodies or PBS served as negative controls and showed no significant change.

**Figure 3:**
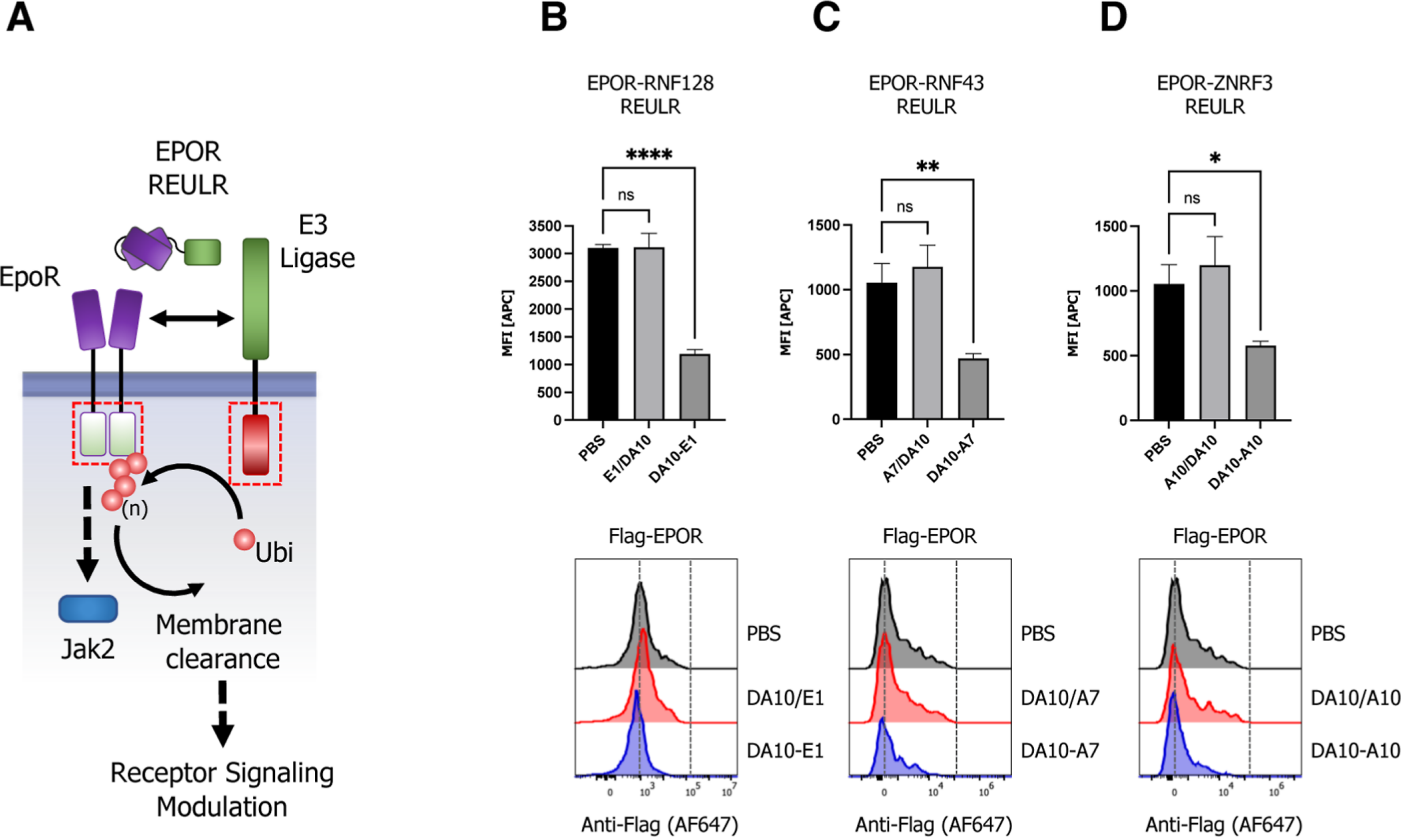
EPOR REULR. (A) Schematic representation of EPOR–REULR-mediated EpoR degradation. (B–D) HEK293T cells were transiently transfected with FLAG-tagged full-length EpoR cDNA (human) under the control of a constitutively active CMV (cytomegalovirus) promoter. 24 h post-transfection, cells were incubated with EPOR–REULR molecules (50 nM) as indicated using RNF128 (E1)-, RNF43 (A7)-, or ZNRF3 (A10)-targeting nanobodies fused to a scFv (single chain fragment variable) reformatted EpoR diabody (DA10). Monomeric binding moieties or PBS were used as a negative control. After 24 h, cells were subjected to FACS analysis using a FLAG antibody (Alexa Fluor 647 conjugate) to monitor EPOR levels on the cell surface. Representative FACS histograms are visualized below the quantified data. Data are mean ± s.d. (*n* = three replicates).

To show modularity with other binding moieties, we reformatted an EpoR-targeting diabody (DA10)^46^ into an scFv (single-chain variable fragment) and fused it to RNF128-, RNF43-, and ZNRF3-targeting nanobodies (Figure 3A). Indeed, intact EPOR–REULR molecules could efficiently degrade EpoR while showing no activity when cells were treated with the monomeric version of the individual targeting arms or PBS (Figures 3B–D and S4A). Of note, the degradation efficiency did not directly correlate with the expression levels of PA-TM-RING E3 ligases observed in HEK293T cells. While RNF128 and RNF43 appear to be expressed at much lower levels than ZNRF3 (∼25×; Figure 1E), degradation using EPOR-RNF128 or EPOR-RNF43 REULR (Figure 3B,C) still resulted in comparable levels of EpoR loss in comparison to a ZNRF3 based EPOR–REULR molecule (Figure 3D).

**Figure 4:**
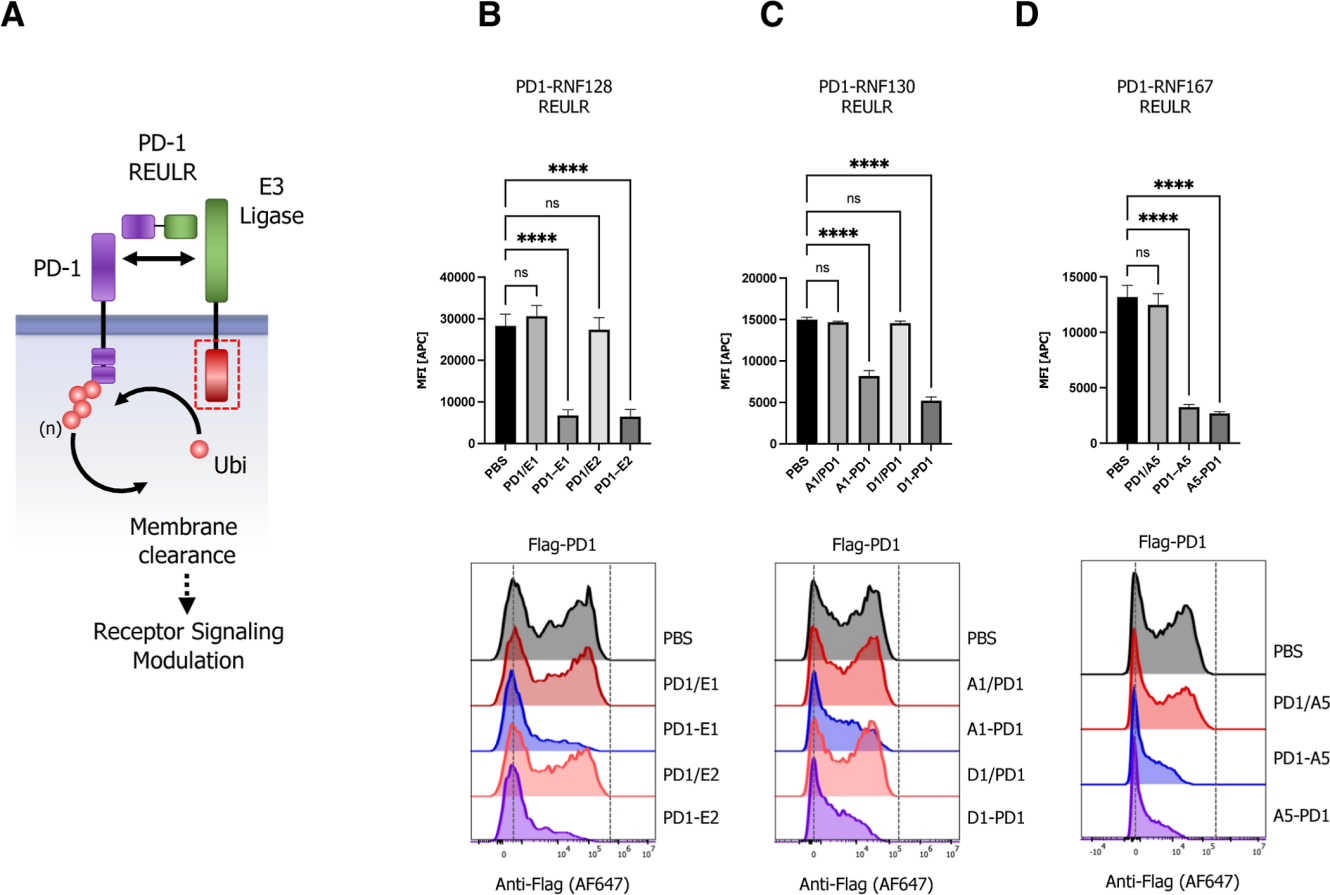
Immune checkpoint REULR. (A) Schematic representation of PD1-REULR-mediated PD-1 degradation. (B–D) HEK293T cells were transiently transfected with FLAG-tagged full-length PD-1 cDNA (human) under the control of a constitutively active CMV (cytomegalovirus) promoter. 24 h post-transfection, cells were incubated with PD1-REULR molecules (50 nM), as indicated using RNF128 (E1; E2)-, RNF130 (A1; D1)-, or RNF167 (A5)-targeting nanobodies fused to a PD-1 binding nanobody (PD1). Monomeric binding moieties or PBS were used as negative controls. After 24 h, cells were subjected to FACS analysis using a FLAG antibody (Alexa Fluor 647 conjugate) to monitor PD-1 levels on the cell surface. Representative FACS histograms are visualized below the quantified data. Data are mean ± s.d. (*n* = three replicates).

Immunotherapies based on checkpoint biology have emerged as a major pillar in fighting cancer. Immune-checkpoint inhibitors (ICIs) such as antibodies targeting CTLA-4 (ipilimumab), PDL1 (atezolizumab and durvalumab), or PD1 (pembrolizumab and nivolumab) have become some of the most widely used anticancer therapies.^47,48^ However, immune-related adverse events (irAEs), such as autoimmune symptoms and tumor hyperprogression, present a significant challenge in the clinic^49^ and a need for the continuous development of immune-oncology pipeline drugs. Targeted protein degradation could provide a major expansion in the repertoire of modulating immune checkpoint receptors by directly regulating their respective cell surface levels. We therefore next generated REULR molecules targeting the immune checkpoint receptor PD-1 (programmed cell death protein 1) by fusing an anti-human PD-1 nanobody^50^ to several nanobodies targeting RNF128, RNF130, and RNF167 (Figure 4A–D). Similar to EGFR– and EPOR–REULRs, treatment of HEK293T cells overexpressing FLAG-tagged full-length PD-1 with a variety of PD1-REULR molecules using RNF128-, RNF130-, or RNF167-targeting nanobodies resulted in a robust and near-complete loss of the PD-1 receptor from the cell surface compared to treatment with the respective monomeric VHHs or PBS (Figures 4B–D and S5A). While RNF130-based REULR molecules worked most effectively in degrading EGFR, PD1-REULR molecules using RNF128 and ENF167 targeting nanobodies collectively resulted in the substantial elimination of PD1.

### Expansion of the REULR Platform to Modulate E3 Ligases Itself: Fratricide REULRs

**Figure 5:**
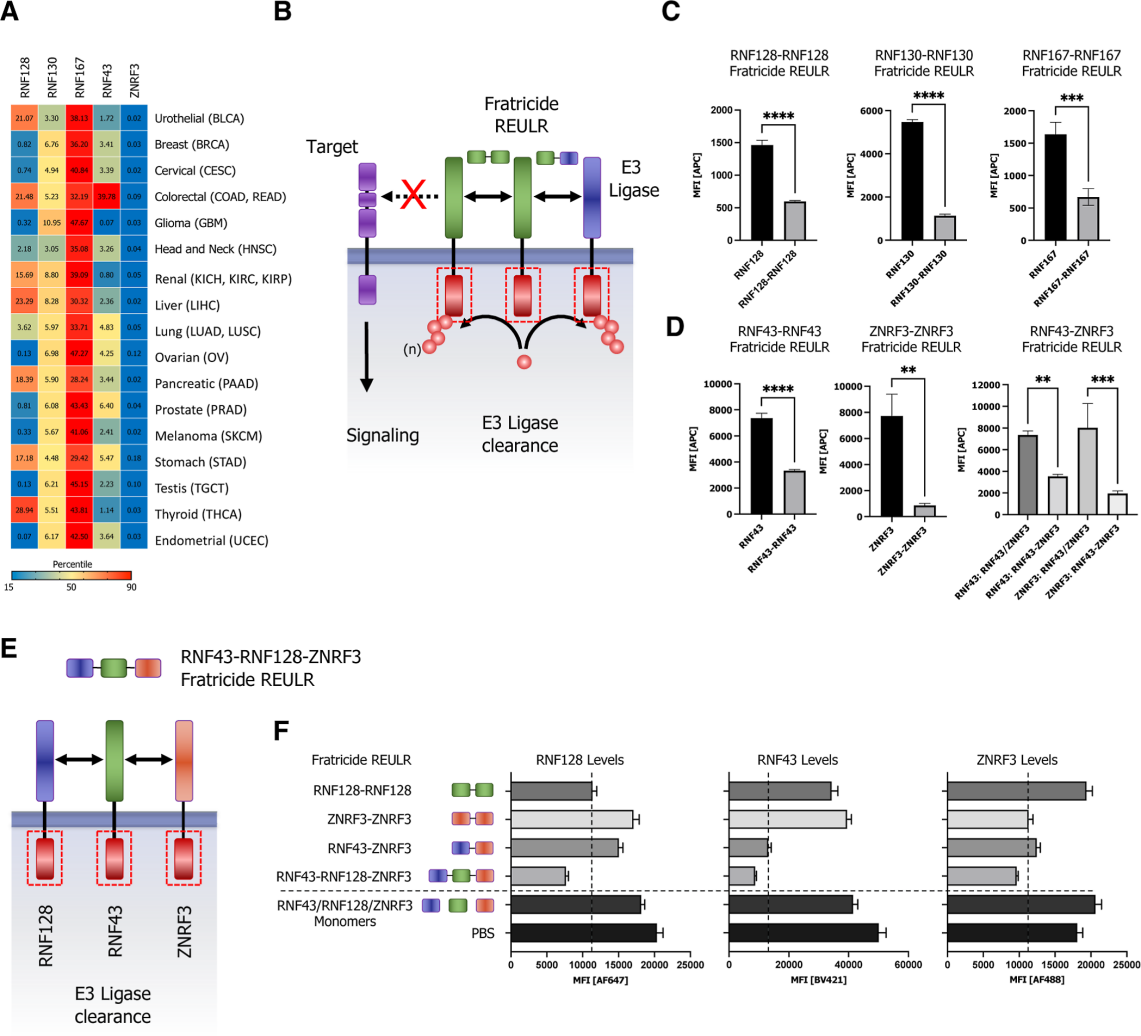
Homo- and heterobifunctional fratricide REULR. (A) TCGA cancer tissue RNA-seq data for RNF128, RNF130, RNF167, RNF43, and ZNRF3 was obtained from 17 cancer types, representing 21 cancer subtypes and were processed as median FPKM (number fragments per kilobase of exon per million reads) and visualized as a hierarchical clustering heatmap. (B) Schematic representation of homo- or heterobispecific fratricide REULR. (C) HEK293T cells were transiently transfected with HA-tagged full-length PA-TM-RING E3 ligase cDNA (human) under the control of a constitutively active CMV (cytomegalovirus) promoter, as indicated. 24 h post-transfection, cells were incubated with fratricide REULR molecules (50 nM), targeting RNF128, RNF130, RNF167, RNF43, or ZNRF3. (D) HEK293T cells were co-transfected with HA-tagged full-length RNF43 and MYC-tagged full-length ZNRF3 cDNA (human) and treated with a heterobispecific RNF43-ZNRF3 fratricide REULR 24 h post-transfection (monomeric binding moieties were used as negative controls). After 24 h, cells were subjected to FACS analysis using a HA antibody (Alexa Fluor 647 conjugate) or an MYC antibody (Alexa Fluor 488 conjugate) to monitor PA-TM-RING E3 ligase levels on the cell surface. Data are mean ± s.d. (*n* = three replicates). (E) Schematic representation of heterobispecific arrayed multimeric fratricide REULR. (F) HEK293T cells were co-transfected with HA-tagged full-length RNF128, MYC-tagged full-length ZNRF3, and FLAG-tagged full-length RNF43 cDNA (human). 24 h post-transfection, cells were treated with homo- or heterobispecific Fratricide REULR molecules as indicated or a RNF43-RNF128-ZNRF3 multimeric fratricide REULR (PBS and monomeric binding moieties were used as negative controls). After 36 h, cells were subjected to FACS analysis using an HA antibody (Alexa Fluor 647 conjugate), a MYC antibody (Alexa Fluor 488 conjugate), and a FLAG antibody (Brilliant Violet 421) to monitor RNF128 (left panel), RNF43 (middle), and ZNRF3 (right panel) E3 ligase levels on the cell surface. Data are mean ± s.d. (*n* = three replicates).

Emerging evidence highlights the pivotal role of RING-type E3 ligases and their substrates in a wide range of human diseases, and mutation of RING-type E3s or modulation of their activity is frequently associated with pathogenesis including viral infections, neurodegenerative disorders, autoimmune diseases, and cancer.^16−18^ Indeed, RNF43 mutations have been associated with aggressive tumor biology such as colorectal and endometrial cancer.^51−53^ To evaluate the impact of other PA-TM-RING E3 ligases in cancer, we analyzed TCGA (The Cancer Genome Atlas) tissue mRNA expression data obtained for RNF128, RNF130, RNF167, RNF43, and ZNRF3 from 17 cancer types, representing 21 cancer subtypes. The data show elevated expression of E3 ligases in various cancer types. Notably, while RNF167 is highly expressed in almost all cancers, other ligases like RNF128 (thyroid, liver, urothelial, and colorectal), RNF130 (gliomas), or RNF43 (colorectal cancers) show a more selective tissue-associated expression pattern (Figure 5A) in cancer cells.

Despite their critical role in regulating protein homeostasis and pathological signaling, our understanding of transmembrane E3 ligase-mediated signaling still remains largely fragmented and can mainly be attributed to the limited availability of tools to study TM E3 ligases. Interestingly, the activity of E3 ligases is tightly regulated by post-translational modifications, and a typical feature of most ligases is the ability to catalyze their own ubiquitination.^54,55^ Based on this paradigm, we postulated that self-regulation by auto-ubiquitination could be used to regulate E3 ligase-dependent signaling.

We therefore proceeded in developing REULR molecules that target the PA-TM-RING E3 ligase itself, either by homodimerization or heterodimerization between two transmembrane E3 ligases. Using this approach would allow strategic modulation of transmembrane E3 ligases and consequently protein homeostasis of their natural targets, a process we termed fratricide REULRs (Figure 5B). Indeed, treatment of cells with RNF128, RNF130, RNF167, RNF43, and ZNRF3 fratricide REULR molecules resulted in an effective loss of cell surface ligase levels in HEK293T cells (Figures 5C–D and S6A).

Furthermore, to demonstrate the modular nature and flexibility of the nanobody-based REULR design, we engineered a RNF43-ZNRF3 heterobifunctional REULR (Figures 5B and S6B) that would allow the elimination of two ligases using one fratricide REULR molecule. The treatment of HEK293T cells overexpressing MYC-tagged RNF43 and HA-tagged ZNRF3 with a RNF43-ZNRF3 heterobifunctional fratricide REULR (Figure 5D; right bar graph) resulted in a significant reduction of RNF43 and a near-compete loss of ZNRF3 levels comparable to RNF43 and ZNRF3 fratricide REULRs (Figure 5D; left and middle bar graph).

To demonstrate the ease of formatting using PA-TM-RING E3-binding VHHs, we extended the previous design into a linear, hetero-trimeric array of VHHs targeting RNF128, RNF43, and ZNRF3 with one fratricide REULR molecule (Figures 5E and S6C). We co-expressed HA-tagged RNF128, MYC-tagged ZNRF3, and FLAG-tagged RNF43 in HEK293T cells and treated cells with RNF128 (only targets RNF128) or ZNRF3-REULR (only targets ZNRF3), heterobifunctional RNF43-ZNRF3 REULR (targets RNF43 and ZNRF3), or a hetero-trimeric RNF43-RNF128-ZNRF3 fratricide REULR that targets all three PA-TM-RING E3 ligases for degradation (Figure 5F). A RNF43-RNF128-ZNRF3-targeting VHH array was able to efficiently eliminate all three E3 ligases from the cell surface and further shows the robustness and advantage of a “mix and match” VHH-based targeting approach.

**Figure 6:**
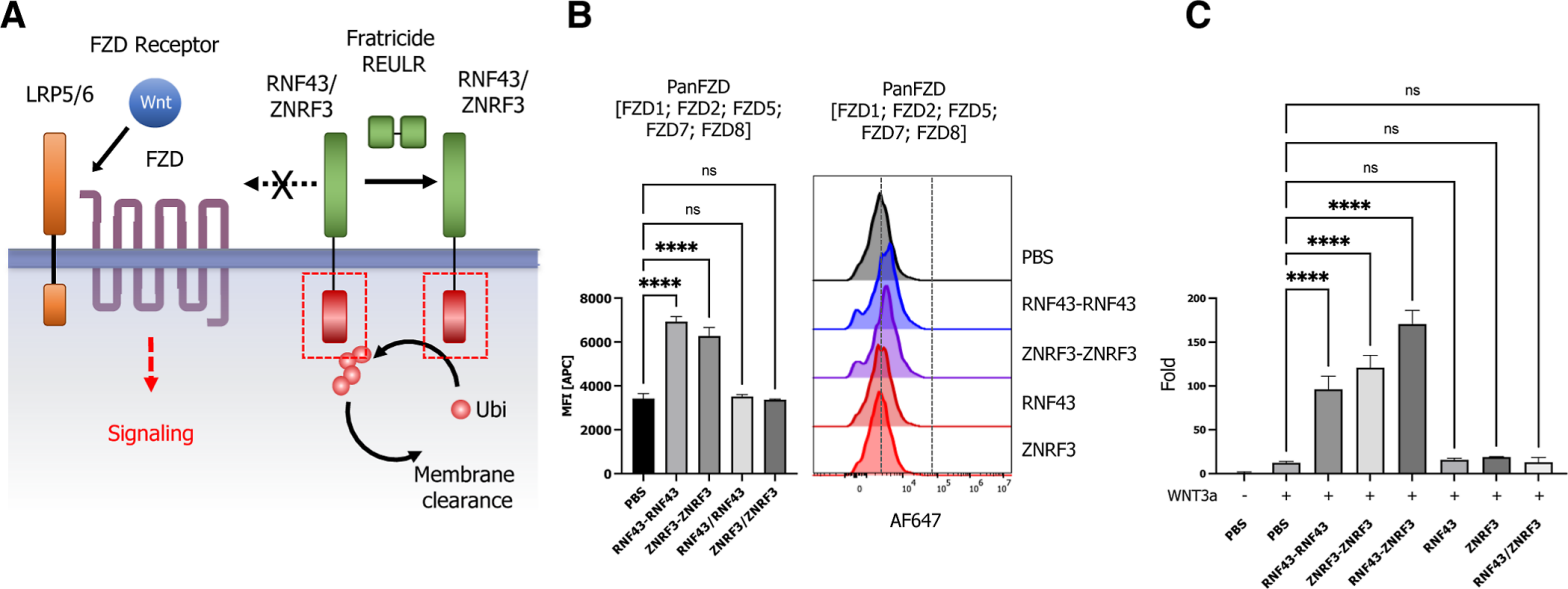
Fratricide REULR and WNT signaling potentiation. (A) Schematic representation of a RNF43- or ZNRF3-based fratricide REULR in the context of a canonical WNT signaling pathway. (B) HEK293STF cells were seeded at 10k/well and subsequently treated with RNF43 or ZNRF3 fratricide REULR for 24 h. FZD cell surface levels were measured by incubating cells with a biotinylated pan-FZD Darpin (DRPB_Fz7/8), recovered by SA647 and analyzed by flow cytometry. Data are mean ± s.d. (*n* = three replicates). (C) HEK293STF cells were seeded at 5k/well and treated with RNF43, ZNRF3, or RNF43-ZNRF3 fratricide REULR molecules after 24 h in the presence of 10% conditioned WNT3a media (monomeric binding moieties or PBS were used as negative controls). After 36 h, the activation of the β-catenin-dependent STF reporter by fratricide REULRs was measured. Data are mean ± s.d. (*n* = three replicates).

The WNT signaling pathway is instrumental for embryonic development, stem cell differentiation, and regeneration of injured tissues, and modulation of WNT signaling presents an untapped potential in regenerative medicine.^56−59^ RNF43 and ZNRF3 are two pivotal PA-TM-RING E3 ligases known to negatively regulate the WNT signaling pathway by targeting Wnt receptors FZD and promoting receptor degradation via the UPS (Figure 6A).^60,61^ With well-established fratricide REULRs in hand, we explored whether RNF43 and ZNRF3-based fratricide REULR molecules have the potential to modulate FZD receptor cell surface levels and potentiate downstream WNT signaling events. We first treated HEK293T cells with RNF43 or ZNRF3 fratricide REULR molecules and monitored FZD cell surface levels using a previously developed pan-FZD (DRPB_Fz7/8) as a staining reagent due to its high affinity and broad binding spectrum for FZD receptors: FZD1, 2, 5, 7, and 8.^62^ We indeed observed a significant increase in the accumulation of FZD levels after RNF43 or ZNRF3 fratricide REULR treatment compared to PBS or monomeric RNF43 or ZNRF nanobodies (Figure 6B). To examine whether these results can be translated into a functional assay and elicit fratricide REULR-specific activation of canonical WNT signaling, we performed a series of reporter assays using HEK 293STF (SuperTopFlash) cells. In agreement with the increased FZD levels upon treatment with RNF43 or ZNRF3 fratricide REULRs, we similarly observed a robust induction of WNT signaling and increased signaling activity using a heterospecific RNF43-ZNRF3 fratricide REULR, compared to treatment with WNT, PBS or monomeric PA-TM-RING nanobodies alone (Figure 6C).

## Discussion

In summary, we implemented a modular, “mix and match” human and mouse cross-reactive nanobody-based targeted protein degradation platform termed REULR by retasking five PA-TM-RING E3 ligases (RNF128, RNF130, RNF167, RNF43, and ZNRF3) to modulate cell surface receptors by induced proximity, allowing selective, tissue-specific application. REULR-based bispecific molecules can be broadly applied to modulate cell surface levels of a variety of therapeutically relevant transmembrane receptors using different binding moieties (Figures 2–4). Furthermore, we present a strategic approach to tune transmembrane E3 ligases itself by using homo-,heterobispecific and arrayed fratricide REULRs and consequently modulate the signaling events of natural target receptors (Figures 5 and 6).

While similar approaches (AbTACs and PROTABs) have been reported to degrade PD-L1 or IGF1R, they are mainly limited by using WNT-responsive E3 ligase RNF43 and ZNRF3^13,14^ and rely on human IgG antibody scaffolds to generate heterobifunctional-targeting molecules. Antibody-derived biologics are generally more constrained by their inherent structural properties including their large size (150 kDa), formatting, and modularity that might limit the applicability for tumor therapy. By contrast, REULR molecules take advantage of the superior pharmacokinetic properties of nanobodies (VHH), allowing for a versatile and modular design with ease of formatting into homo- or heterobifunctional dimers or arrayed multimers to target one or multiple cell surface proteins (Figures 2–5). While nanobodies have been proven to possess a low immunogenicity risk profile, it is important to consider that they can show limitations in their therapeutic lifetime due to rapid renal clearance without further engineering, e.g., half-life extension through the use of serum albumin nanobody fusions (NbHSA) which could be achieved due to the modular nature of the REULR molecules.^63^

Interestingly, we observed that the affinity and expression levels of the PA-TM-RING E3 ligase-targeting nanobody did not directly correlate with the levels of cell surface clearance. This suggests that the PA-TM-RING E3 ligases operate with a wide spectrum of cytosolic catalytic RING E3 activity rather than by abundance alone. Catalytic activity and processivity of E3 ligases are regulated by many contributing factors to safeguard substrate selection including cell-type expression levels and tightly regulated post-translational modifications including phosphorylation and sumoylation among others, as well as binding ubiquitin proteasome machinery adapters.^15^ Furthermore, E3 ligase protein homeostasis is regulated by ubiquitylation itself and subsequent internalization; thus, there is likely a pool of E3 ligase whose activity levels are unknown.^55,64^ Moreover, REULR processivity may be further influenced by the orientation and geometry of the ternary receptor–REULR–ligase complex.

In addition to the therapeutic potential of REULR molecules, the monomeric-binding modules (nanobodies) themselves present invaluable tools to validate natural targets and to gain a deeper understanding into the fundamental biological function of transmembrane E3 ligases and their cellular pathways in drug discovery and the context of cancer biology.

Collectively, we believe that our “mix and match” nanobody-based REULR protein degradation strategy holds tremendous promise for a large variety of targets and serves as a powerful research tool with the potential to develop novel therapeutic applications that can be easily customized by virtue of its modularity, human and mouse cross-reactivity, and tissue specificity.

## Materials and Methods

### Curation of the Human Ubiquitin Cell Surface Receptor Proteome

A raw list of reported ubiquitin sites was obtained from PhosphoSitePlus (PSP; https://www.phosphosite.org) and matched to a curated list of the human membrane proteome^65^ to generate a master list of cell surface receptors with reported ubiquitination sites.

### Database Integration

Pairwise protein sequence alignments were performed using the Smith–Waterman algorithm to calculate alignments between human and mouse amino acid sequences obtained from UniProt (https://www.uniprot.org/). Phylogenetic homology analysis was performed to generate phylogenetic trees from multiple sequence alignments (MSA) of amino acid sequences of ECD sequences of transmembrane cell surface receptors (https://www.uniprot.org/). Briefly, MSA was performed using ClustalOmega (https://www.ebi.ac.uk/Tools/msa/clustalo/), and alignment results were submitted to calculate phylogenetic tree parameters (https://www.ebi.ac.uk/Tools/phylogeny/simple phylogeny/), which were visualized by Interactive Tree of Life (iTOL; https://itol.embl.de/).^66^ Tissue expression datasets and normal tissue and TCGA datasets were downloaded from The Human Protein Atlas (https://www.proteinatlas.org; v21.1). TCGA cancer tissue RNA-seq data were obtained from 17 cancer types, representing 21 cancer subtypes, and were processed as median FPKM (number of fragments per kilobase of exon per million reads) and visualized as a hierarchical clustering heatmap using JMP Pro (v16). Unsupervised hierarchical clustering of normalized mRNA gene expression by tissue was performed with the Ward linkage, and correlation distances were plotted as heatmaps using JMP Pro (v16).

### Cell Lines

Suspension cells were grown in plain-bottom, vented flasks (Thermo); adherent cells were grown in T25 or T75 flasks (ThermoFisher). Cells were maintained at 37 °C and 5% CO_2_. HEK293T (CRL-3216; ATCC), and LentiX cells were maintained in DMEM supplemented with 10% FBS, 1% GlutaMax, and 1% penicillin/streptomycin. Caco-2, YT1, A431, UT/7, BaF3, 3T3, and B16 cells were obtained from ATCC and grown and maintained according to ATCC specifications. HEK293F (R79007; ThermoFisher) were grown in FreeStyle media (12338018; ThermoFisher). Expi293F (A14528; ThermoFisher) cells were grown in Expi293 Expression Medium (ThermoFisher). Cell lines tested negative for mycoplasma (MycoAlert Mycoplasma Detection kit, Lonza).

### Facs Staining

Cells were stained with the indicated antibodies at a 1:100 dilution or tetramer at the indicated concentration for 30 min on ice in MACS staining buffer (Miltenyi). After incubation with fluorescent antibodies or tetramers, cells were washed with MACS buffer and analyzed via flow cytometry on a Cytoflex (Beckman Coulter) instrument. Surface expression was quantified by FACS using the CytoFLEX, equipped with a high-throughput sampler. Live cells were identified after gating on the basis of forward scatter (FSC) and side scatter (SSC) and propidium iodide (PI)-negative staining. Data were analyzed using FlowJo 10.8.1 (BD). All assays were performed using independent biological replicates. The number of replicates (*n*) is indicated in the figure legends. The mean fluorescence intensity (MFI) was determined in FlowJo 10.8.1.

## Antibodies

Primary antibodies used in this study include the anti-DYKDDDDK tag (CST, D6W5B, no. 15009), anti-HA Tag (CST, 6 × 10^2^, no. 3444), and anti-MYC (CST, 9B11, no. 2279). Antibodies were used at 1:100 dilution in MACS staining buffer (Miltenyi).

### Production of Purified Proteins

Proteins were produced in Expi293F cells using transfection conditions following the manufacturer’s protocol. After harvesting of cell media, 1 M Tris, pH 8.0 was added to a final concentration of 20 mM. Ni-NTA agarose (Qiagen) was added to ∼5% media volume. 1× sterile PBS, pH 7.2 (Gibco) was added to ∼3× medium volume. The mixture was stirred overnight at 4 °C. Ni-NTA agarose beads were collected in a Buchner funnel and washed with ∼300 mL protein wash buffer (20 mM HEPES, pH 7.2, 150 mM NaCl, 20 mM imidazole). Beads were transferred to an Econo-Pak chromatography column (Bio-Rad), and the protein was eluted in 15 mL of elution buffer (20 mM HEPES, pH 7.2, 150 mM NaCl, 200 mM imidazole). The DNA encoding for pan-FZD (DRPB_Fz7/8) was cloned into pET-28 with a C-terminal AVI-6xHIS tag and transformed into Rosetta DE3-competent cells. The cells were grown at 37 °C in 2YT media supplemented with kanamycin (40 μg/mL) until the culture reached log-phase growth. IPTG was added to the culture to induce protein expression at a final concentration of 1 mM. The culture was shaken at 37 °C for 3 h, and protein was harvested from the cells by sonication. Pan-Fzd protein was purified using Ni-NTA agarose (Qiagen), followed by biotinylation and size-exclusion chromatography with a Superdex S75 column (GE Healthcare). In general, proteins were concentrated using Amicon Ultracel filters (Millipore), and absorbance at 280 nm was measured using a Nanodrop 2000 spectrophotometer (Thermo Fisher Scientific) to determine protein concentrations.

### REULR Design and Expression

All proteins were cloned in-frame in a modified pD649 plasmid with a N-terminal hemagglutinin signal peptide (HAsp) and a C-terminal AVI-6xHIS tag for protein expression and purification from Expi293F cells. REULR molecules were connected either by a LEVLFQGP (3C) or a GSLEVLFQGPGS (GS flanked 3C) linker. All VHH and scFv sequences were cloned using gBlocks (IDT), and final sequence integrity was confirmed by DNA sequencing. All amino acid sequences can be found in Table S2A–C.

### Biotinylation and FPLC Purification

Where indicated, proteins were biotinylated as described previously.^67^ Briefly, up to 10 mg of protein was incubated at 4°C overnight in 2× Biomix A (0.5 M bicine buffer), 2× Biomix B (100 mM ATP, 100 mM MgOAc, 500 μM d-biotin), and Bio200 (500 μM d-biotin) to a final concentration of 20 μM, and 60–80 units BirA ligase in a final volume of 1 mL. All proteins were further purified by size-exclusion chromatography using an S75 or a S200 Increase column (GE Healthcare), depending on protein size, on an ÄKTA Pure FPLC (GE Healthcare).

### Nanobody Selection

Nanobody selection was performed as previously described with minor alterations. Briefly, the synthetic yeast library was expanded overnight in -Trp media with glucose at 30 °C and induced at 10× the theoretical diversity by suspension in -Trp media with galactose, grown at 20 °C for 24 h. Surface display was assessed by flow cytometry after staining with an anti-HA antibody. Rounds 1 and 2 were first negatively selected on magnetic streptavidin beads and then positively selected on magnetic streptavidin beads loaded with biotinylated target protein. Subsequent rounds were carried out with target proteins tetramerized by streptavidin and bound to anti-fluorophore magnetic beads, followed by decreasing monomer protein concentrations and by flow cytometry. Single clones from the final round were sorted into 96 well plates, induced for 24 h at 20 °C, and grown in deep well blocks. The top 20 clones were sequenced, and unique clones were expressed in Expi293F cells and assayed for binding to the corresponding target protein by SPR.

### SPR Experiments

SPR experiments were performed using a Biacore T100 instrument (GE Healthcare). FPLC-purified biotinylated proteins (ligands) in HBS-P + buffer (GE Healthcare) were captured on a streptavidin (SA) series S sensor chip (GE Healthcare). Chip capture was performed in HBS-P + buffer (GE Healthcare) to aim for ∼100–200 ligand response units (RU). Flow cell 1 was left empty as a reference flow-cell for on-line subtraction of bulk solution refractive index and for evaluation of non-specific binding of the analyte to the chip surface using Biacore T100 Control Software (version 3.2) (GE Healthcare). FPLC-purified non-biotinylated protein was used as the analyte. Analytes were run in HBS-P + buffer using twofold increasing protein concentrations to generate a series of sensograms. Binding parameters were either determined based on a 1:1 Langmuir model or at equilibrium using the accompanying Biacore T100 evaluation software. A table of all SPR conditions for each ligand–analyte pair tested including the concentration range of twofold analyte dilutions, injection rate, injection and dissociation times, and regeneration conditions can be found in Table S1. FPLC traces for purified proteins used for SPR can be found in Figure S1.

### Cell–Surface Binding Assay with Streptavidin-Tetramerized Proteins

To examine PPIs at the cell surface, we performed cell–surface protein binding assays using human or mouse cell lines, or primary cells (PBMCs) with streptavidin-tetramerized, biotinylated proteins. To generate streptavidin-tetramerized proteins to test for binding to cells, FPLC-purified biotinylated proteins (see above) were incubated with streptavidin tetramers conjugated to Alexa647 Fluor (SA-647) (Thermo Fisher) at a 4:1 molar ratio on ice for at least 15 min. Approximately 150,000 cells were incubated with protein:SA-647 complexes in a final volume of 100 μL in 96-well round-bottom plates (Corning) for 30-60 min at 4 °C protected from light. Following incubation, cells were washed two times with 200 μL cold MACS buffer and resuspended in 200 μL cold MACS buffer with 1:3000 PI (Thermo Fisher Scientific). Immunofluorescence staining was analyzed using a Cytoflex (Beckman Coulter), and data were collected for 20,000 cells. Data were analyzed using FlowJo v10.4.2 software. All data report MFI. Concentration-dependent binding of protein:SA-647 to full-length receptor-expressing, but not mock control cells, was deemed indicative of cell–surface binding.

### STF Luciferase Reporter Assays

HEK293STF cells were seeded for each condition in 96-well plates and stimulated with fratricide REULRs, WNT (WNT3a conditioned media; ATCC), control proteins, or PBS for 36 h. After washing cells with 1× PBS, cells in each well were lysed in 30 μL 1× passive lysis buffer (Promega). 10 μL per well of lysate was assayed using the Dual Luciferase Assay kit (Promega).

### Cell Proliferation Assay

A431 cells were seeded at 2.5k cell/well. After 24 h, cells were treated with PBS, Cetuximab, or different EGFR–REULR molecules (50 nM). Cells were incubated for 72 h, washed, and subjected to CellTiter-Glo (2.0) assays to measure cell proliferation, according to the manufacturer’s specifications (Promega). Data are presented as a percentage of untreated cells (*n* = 4).

### Statistics

All figures are representative of at least *n* = 3 (in vitro) experiments, unless otherwise noted. Statistical significance was assayed by grouped, one-way ANOVA using GraphPad Prism 9.4.1. In all figures, *P < 0.05; **P < 0.01; ***P < 0.001; ****P < 0.0001; NS: not significant. Data are represented as mean ± s.d., unless otherwise stated.

## Data Availability

All data generated or analyzed during this study are included in the manuscript and supporting files.

## Supporting Information Available

Size-exclusion chromatography, SPR sensograms, SPR conditions, pairwise sequence alignments, cell surface staining gating strategies, REULR design, REULR architecture, and REULR-related amino acid sequences (PDF)
