# Supplementary material for: Receptor Elimination by E3 Ubiquitin Ligase Recruitment (REULR): A Targeted Protein Degradation Toolbox
Source: ACS Synth Biol. 2023 Apr 3;12(4):1081–93. doi: 10.1021/acssynbio.2c00587 (PMC10127277; doi:10.1021/acssynbio.2c00587)
Supplement: Supplementary file 1 — sb2c00587_si_001.pdf [file sb2c00587_si_001.pdf]

Supporting Information

Receptor Elimination by E3 Ubiquitin Ligase Recruitment (REULR): A Targeted Protein Degradation Toolbox.

Dirk H. Siepe[1], Lora K. Picton[1], K. Christopher Garcia[1,2,3*]

[1]Department of Molecular and Cellular Physiology, Stanford University School of Medicine, Stanford, CA 94305, USA.
[2]Department of Structural Biology, Stanford University School of Medicine, Stanford, CA 94305, USA.
[3]Howard Hughes Medical Institute, Stanford University School of Medicine, Stanford, CA 94305, USA.

[*]Lead Contact. kcgarcia@stanford.edu

Figure S1

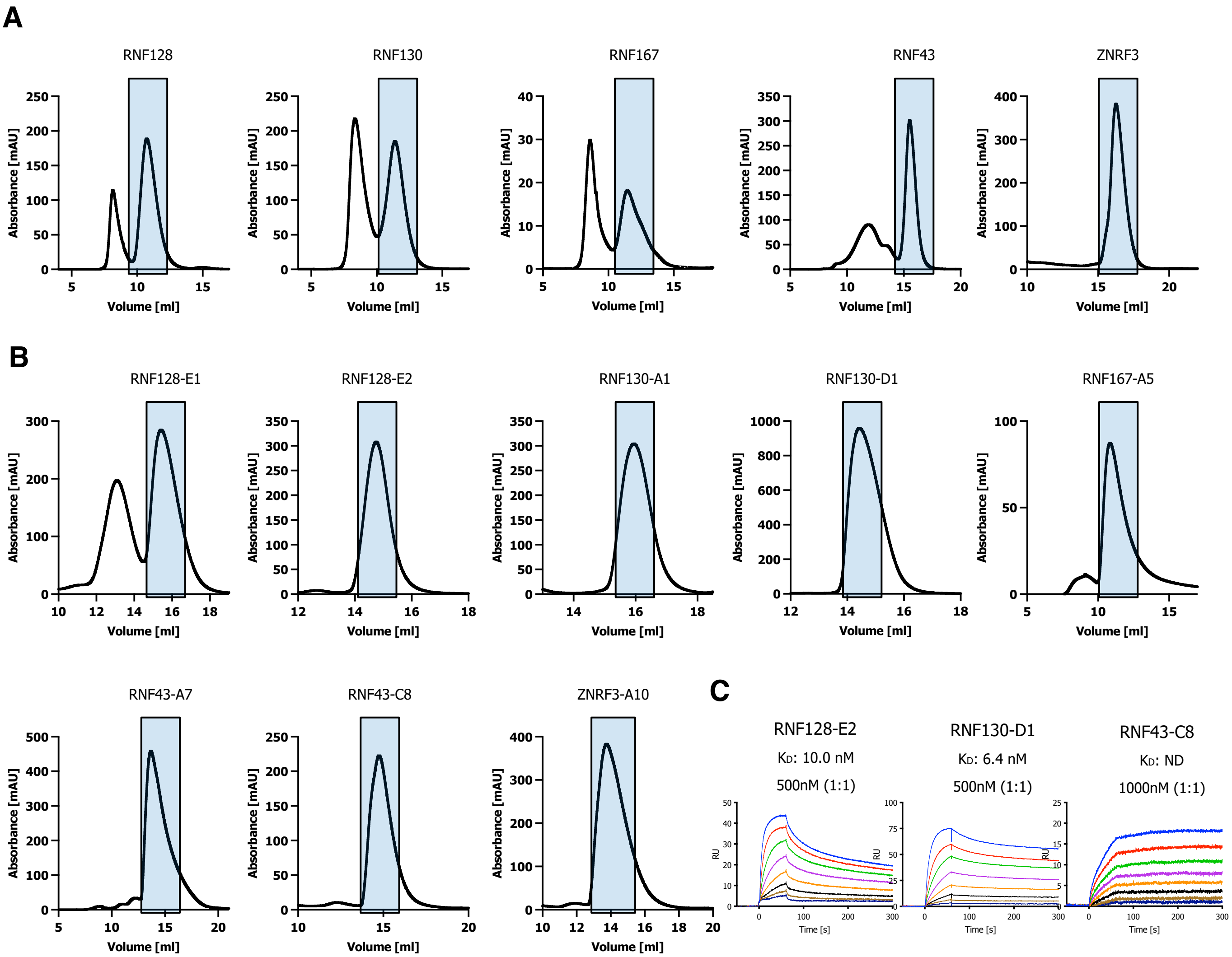

D

Human/Mouse	Similarity	Identity	Gaps
RNF128	97.95	96.15	0.00
RNF130	99.74	98.72	0.00
RNF167	96.3	93.83	0.62
RNF43	95.98	91.38	0.00
ZNRF3	98.78	98.17	0.00
**Average [%]**	**97.75**	**95.65**	**0.12**

Figure S1

Figure S1. Size-Exclusion Chromatography, SPR sensograms and pairwise sequence alignments.

(A, B) Size-Exclusion Chromatography traces of RNF128, RNF130, RNF167, RNF43 and ZNRF3 PA-TM-RING E3 Ligase ECDs used for screening the synthetic nanobody library and SPR validation of PA-TM-RING E3 Ligase VHHs. (C) SPR sensograms and binding affinities of PA-TM-RING ligase selected nanobodies (analytes) for human RNF128, RNF130 and RNF43 ECDs (ligands). (D) Pairwise protein sequence alignments of PA-TM-RING E3 Ligases were performed using Smith-Waterman algorithm to calculate alignments between human and mouse amino acid sequences.

Figure S2

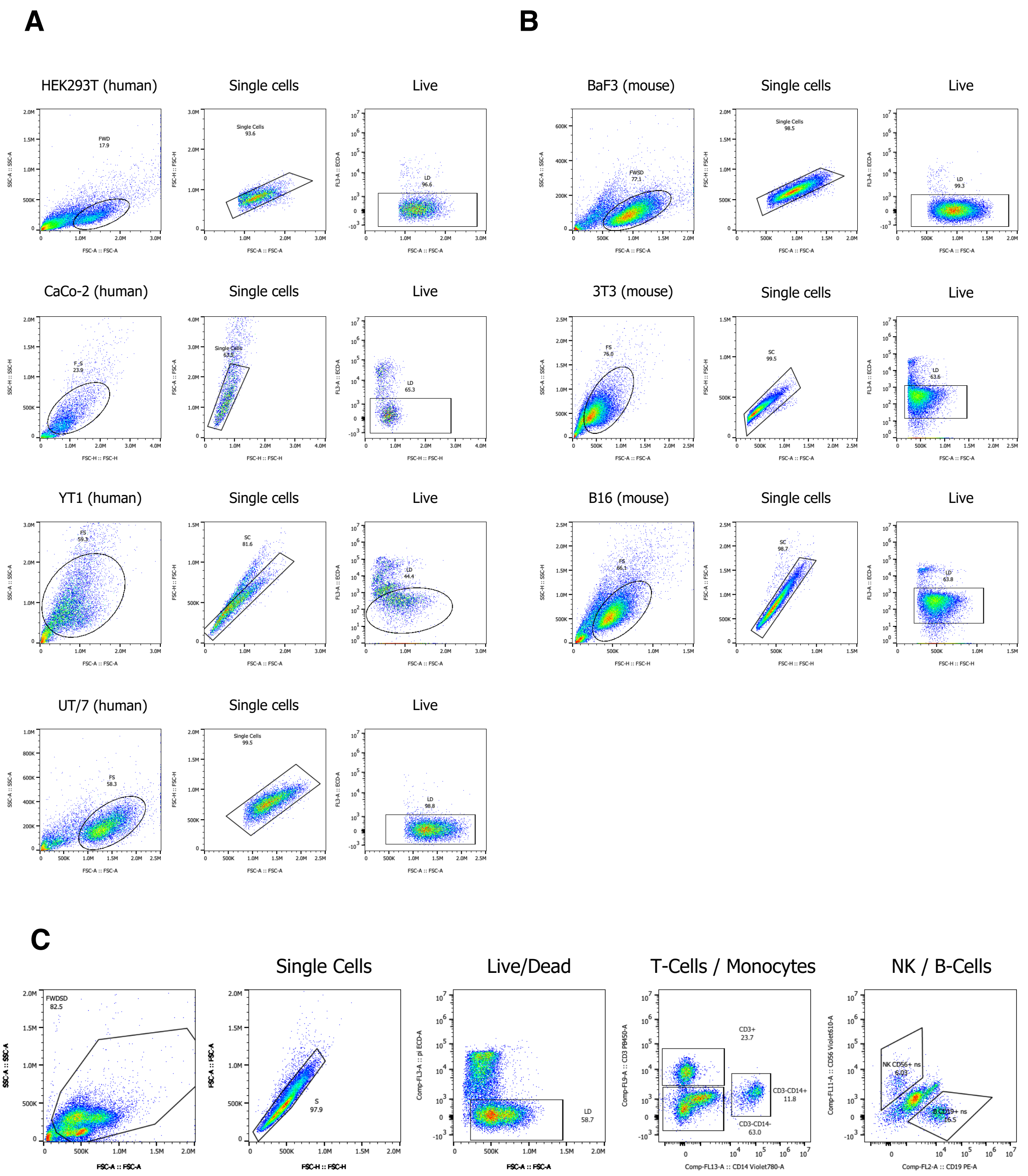

A
B
HEK293T (human)
Single cells
Live
BaF3 (mouse)
CaCo-2 (human)
3T3 (mouse)
YT1 (human)
B16 (mouse)
UT/7 (human)
C
Single Cells
Live/Dead
T-Cells / Monocytes
NK / B-Cells
FWDSD
82.5
S
97.9
LD
58.7
CD3+
23.7
CD3-CD14+
11.8
CD3-CD14-
63.0
NK CD56+ ns
6.03
B CD19+ ns
16.5
FSC-A :: FSC-A
SSC-A :: SSC-A
FSC-H :: FSC-H
Comp-FL3-A :: pI ECD-A
Comp-FL9-A :: CD3 PB450-A
Comp-FL13-A :: CD14 Violet780-A
Comp-FL11-A :: CD56 Violet610-A
Comp-FL2-A :: CD19 PE-A

Figure S2

Figure S2. Cell Surface Staining Gating strategies.

(A) Gating strategy of human HEK293T, CaCo-2, YT1, UT/7 cell lines. (B) Gating strategy of mouse BaF3, 3T3, B16 cell lines. (C) Gating strategy of Primary Peripheral Blood Mononuclear Cells (PBMCs).

Figure S3

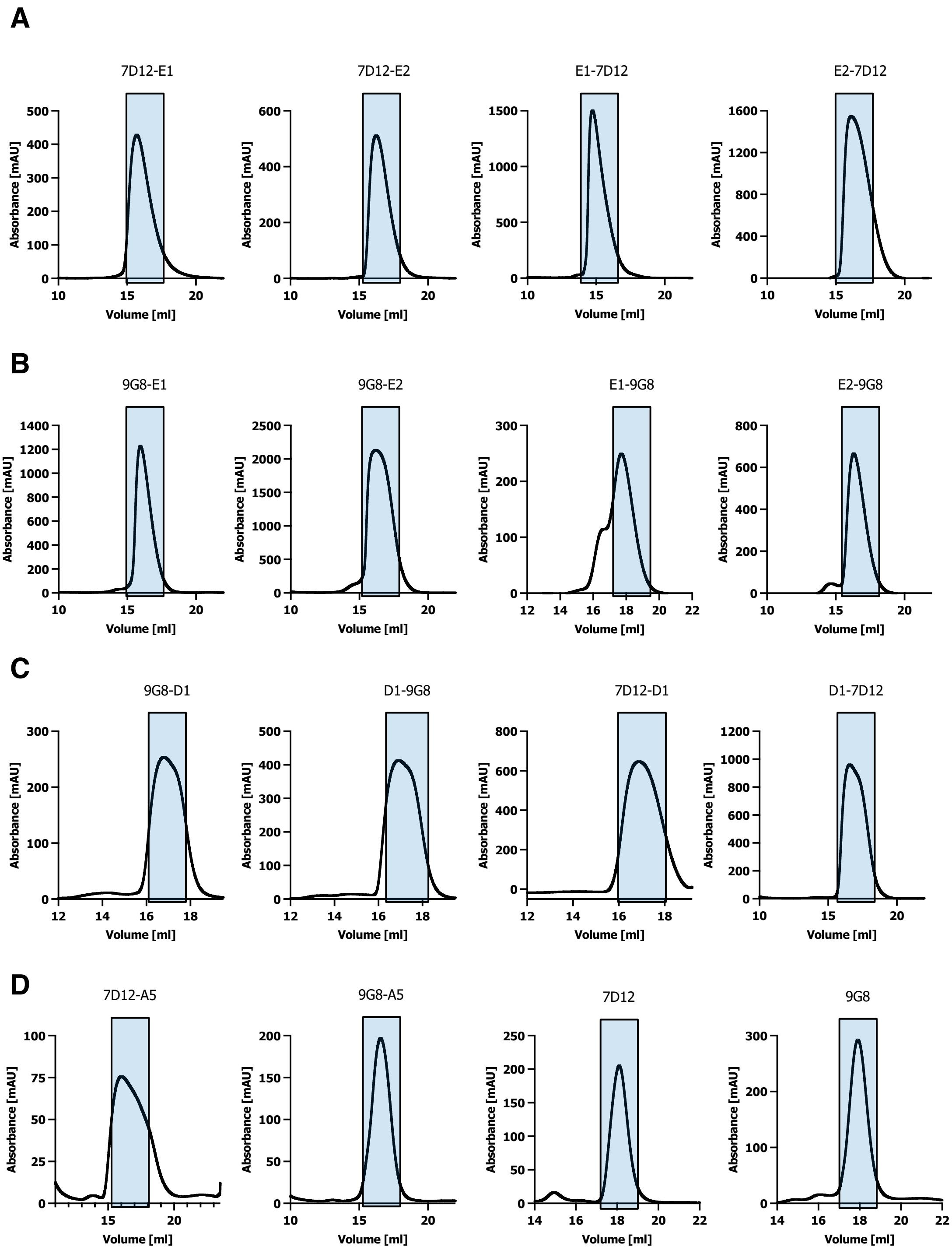
A
7D12-E1
7D12-E2
E1-7D12
E2-7D12
B
9G8-E1
9G8-E2
E1-9G8
E2-9G8
C
9G8-D1
D1-9G8
7D12-D1
D1-7D12
D
7D12-A5
9G8-A5
7D12
9G8
Absorbance [mAU]
Volume [ml]

Figure S3

Figure S3. Size-Exclusion Chromatography.

(A-D) Size-Exclusion Chromatography traces of proteins used for EGFR-REULR experiments.

Figure S4

A

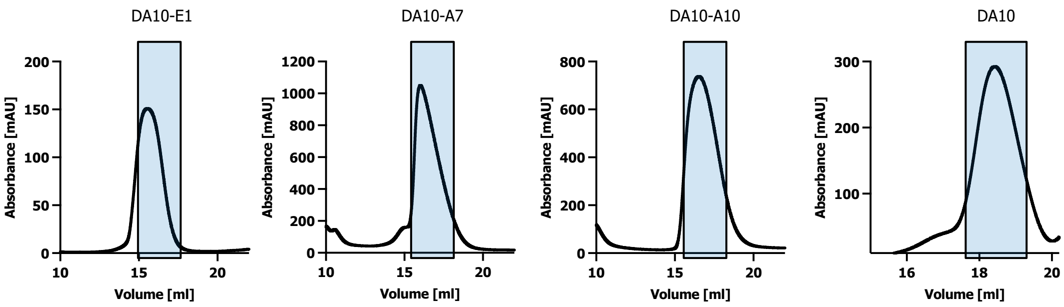

DA10-E1
DA10-A7
DA10-A10
DA10
Absorbance [mAU]
Volume [ml]
0
50
100
150
200
200
400
600
800
1000
1200
300
10
15
20
16
18

Figure S4

Figure S4. Size-Exclusion Chromatography.

(A) Size-Exclusion Chromatography traces of proteins used for EPOR-REULR experiments.

Figure S5

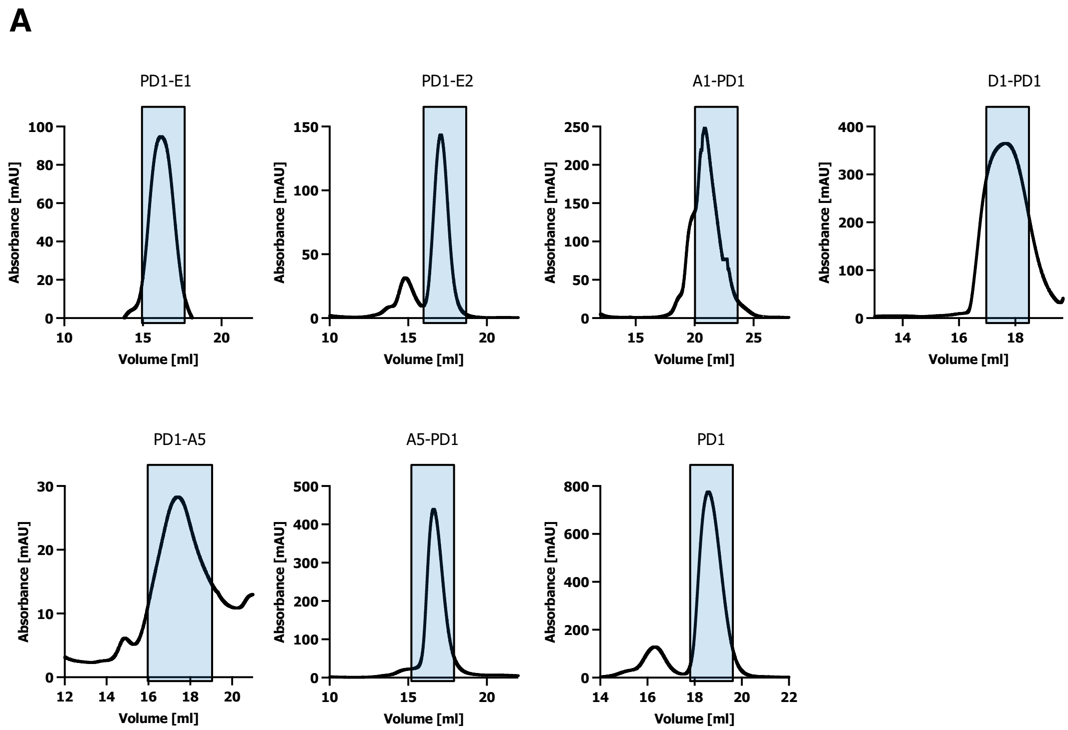
A
PD1-E1
PD1-E2
A1-PD1
D1-PD1
PD1-A5
A5-PD1
PD1
Absorbance [mAU]
Volume [ml]

Figure S5

Figure S5. Size-Exclusion Chromatography.

(A) Size-Exclusion Chromatography traces of proteins used for PD1-REULR experiments.

Figure S6

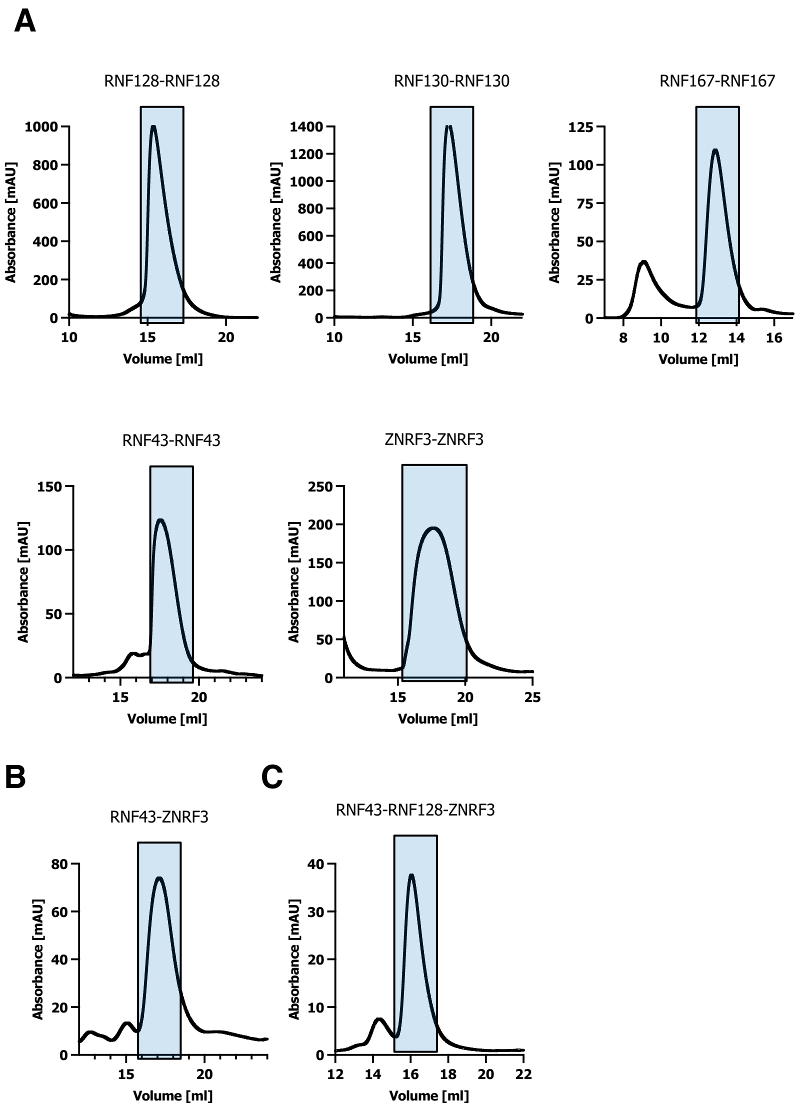
A
RNF128-RNF128
RNF130-RNF130
RNF167-RNF167
RNF43-RNF43
ZNRF3-ZNRF3
B
RNF43-ZNRF3
C
RNF43-RNF128-ZNRF3
Absorbance [mAU]
Volume [ml]

Figure S6

Figure S6. Size-Exclusion Chromatography.

(A-C) Size-Exclusion Chromatography traces of proteins used for Fratricide REULR experiments.

Table S1

A

Ligand (bio+)	Analyte (bio-)	Max Analyte (nM)	Dilution Series	Injection (sec)	Injection (ul/min)	Dissociation (sec)	Regeneration	Regen (ul/min)
RNF128 ECD	RNF128 E1	500	2	60	30	240	1 x 10 sec Glycin pH 2.5	50
RNF128 ECD	RNF128 E2	500	2	60	30	240	1 x 10 sec Glycin pH 2.5	50
RNF130 ECD	RNF130 A1	500	2	60	30	240	1 x 10 sec Glycin pH 2.5	50
RNF130 ECD	RNF130 D1	500	2	60	30	240	1 x 10 sec Glycin pH 2.5	50
RNF167 ECD	RNF167 A5	1000	2	60	30	240	1 x 10 sec Glycin pH 2.5	50
RNF43 ECD	RNF43 A7	1000	2	60	30	240	1 x 10 sec Glycin pH 2.5	50
RNF43 ECD	RNF43 C8	1000	2	60	30	240	1 x 10 sec Glycin pH 2.5	50
ZNRF3 ECD	ZNRF3 A10	125	2	60	30	240	1 x 10 sec Glycin pH 2.5	50

Table S1

Table S1. SPR Conditions.

(A) Table of SPR conditions for all ligand-analyte pairs tested including ligand RU, maximum analyte concentration, analyte RU at maximum concentration, injection time (seconds), injection rate (ul/minute), dissociation time (seconds) and regeneration conditions.

Table S2

A

NAME	TYPE	FORMAT	DESIGN	AA SEQUENCE	#
RNF128-E1	VHH	Monomer	RNF128	QVQLQESGGGLVQAGGSLRLSCAASGNISVQLDMGWYRQAPGKEREFVAAINQGTTTYYADSVKGRFTISR<br>DNAKNTVYLQMNSLKPEDTAVYYCAVYLYDIWNHPYWGQGTQVTVSS	118
RNF128-E2	VHH	Monomer	RNF128	QVQLQESGGGLVQAGGSLRLSCAASGSISGGKGMGWYRQAPGKEREFVAAIGSGAITYYADSVKGRFTISR<br>DNAKNTVYLQMNSLKPEDTAVYYCAVYTTALDEYPYWGQGTQVTVSS	118
RNF130-A1	VHH	Monomer	RNF130	QVQLQESGGGLVQAGGSLRLSCAASGYISGYYVMGWYRQAPGKEREFVASISYGASTYYADSVKGRFTISR<br>DNAKNTVYLQMNSLKPEDTAVYYCAVDFDSNYAHTYWGQGTQVTVSS	118
RNF130-D1	VHH	Monomer	RNF130	QVQLQESGGGLVQAGGSLRLSCAASGTISFIGYMGWYRQAPGKERELVASIASGTSTYYADSVKGRFTISRD<br>NAKNTVYLQMNSLKPEDTAVYYCAATQYIQDVHRYWGQGTQVTVSS	118
RNF167-A5	VHH	Monomer	RNF167	QVQLQESGGGLVQAGGSLRLSCAASGSIFRLWYMGWYRQAPGKEREFVASIGIGATTNYADSVKGRFTISR<br>DNAKNTVYLQMNSLKPEDTAVYYCAVFGWAYSGYHDDFLYWGQGTQVTVSS	122
RNF43-A7	VHH	Monomer	RNF43	QVQLQESGGGLVQAGGSLRLSCAASGTIFGLSDMGWYRQAPGKEREFVAAIAGGTITYYADSVKGRFTISR<br>DNAKNTVYLQMNSLKPEDTAVYYCAAYKYDERYHSYWGQGTQVTVSS	118
RNF43-C8	VHH	Monomer	RNF43	QVQLQESGGGLVQAGGSLRLSCAASGSIFWKPVMGWYRQAPGKEREFVAAITSGTNTYYADSVKGRFTISR<br>DNAKNTVYLQMNSLKPEDTAVYYCAVDDYDVVEYPYWGQGTQVTVSS	118
ZNRF-A10	VHH	Monomer	ZNRF3	QVQLQESGGGLVQAGGSLRLSCAASGTISYAHIMGWYRQAPGKERELVAGISQGSITNYADSVKGRFTISRD<br>NAKNTVYLQMNSLKPEDTAVYYCAVISYDYIKSVPFRYWGQGTQVTVSS	121
7D12	VHH	Monomer	EGFR	QVKLEESGGGSVQTGGSLRLTCAASGRTSRSYGMGWFRQAPGKEREFVSGISWRGDSTGYADSVKGRFTI<br>SRDNAKNTVDLQMNSLKPEDTAIYYCAAAAGSAWYGTLYEYDYWGQGTQVTVSS	124
9G8	VHH	Monomer	EGFR	EVQLVESGGGLVQAGGSLRLSCAASGRTFSSYAMGWFRQAPGKEREFVVAINWSSGSTYYADSVKGRFTI<br>SRDNAKNTMYLQMNSLKPEDTAVYYCAAGYQINSGNYNFKDYEYDYWGQGTQVTVSS	127
DA10	scFv	Monomer	EPOR	EVQLLESGGGLVQPGGSLRLSCAASGFTFSSYAMSWVRQAPGKGLEWVSAISGSGGSTYYADSVKGRFTIS<br>RDNSKNTLYLQMNSLRAEDTAVYYCVKDRVAVAGKGSYYFDSWGRGTTVTVSSGGGGSGGGGSGGGGS<br>QSVLTQPPSVSEAPGQRVTIACSGSSSNIGNNAVSWYQQLPGKAPTLLIYYDNLLPSGVSDRFSGSKSGTSA<br>SLAISGLQSEDEADYYCAAWDDSLNDWVFGGGTKVTVL	249
PD1	VHH	Monomer	PD1	DVQLVESGGGVVQPGGSLRLSCAASGSIASIHAMGWFRQAPGKEREFVAVITWSGGITYYADSVKGRFTISR<br>DNSKNTVYLQMNSLRPEDTALYYCAGDKHQSSWYDYWGQGTLVTVSS	119

Table S2

B

NAME	TYPE	FORMAT	DESIGN	AA SEQUENCE	#
7D12-E1	VHH-VHH	Dimer	EGFR-RNF128	QVKLEESGGGSVQTGGSLRLTCAASGRTSRSYGMGWFRQAPGKEREFVSGISWRGDSTGYADSVKGRFTI<br>SRDNAKNTVDLQMNSLKPEDTAIYYCAAAAGSAWYGTLYEYDYWGQGTQVTVSSLEVLFQGPQVQLQESG<br>GGLVQAGGSLRLSCAASGNISVQLDMGWYRQAPGKEREFVAAINQGTTTYYADSVKGRFTISRDNAKNTVY<br>LQMNSLKPEDTAVYYCAVYLYDIWNHPYWGQGTQVTVSS	250
7D12-E2	VHH-VHH	Dimer	EGFR-RNF128	QVKLEESGGGSVQTGGSLRLTCAASGRTSRSYGMGWFRQAPGKEREFVSGISWRGDSTGYADSVKGRFTI<br>SRDNAKNTVDLQMNSLKPEDTAIYYCAAAAGSAWYGTLYEYDYWGQGTQVTVSSLEVLFQGPQVQLQESG<br>GGLVQAGGSLRLSCAASGSISGGKGMGWYRQAPGKEREFVAAIGSGAITYYADSVKGRFTISRDNAKNTVY<br>LQMNSLKPEDTAVYYCAVYTTALDEYPYWGQGTQVTVSS	250
E1-7D12	VHH-VHH	Dimer	EGFR-RNF128	QVQLQESGGGLVQAGGSLRLSCAASGNISVQLDMGWYRQAPGKEREFVAAINQGTTTYYADSVKGRFTISR<br>DNAKNTVYLQMNSLKPEDTAVYYCAVYLYDIWNHPYWGQGTQVTVSSLEVLFQGPQVKLEESGGGSVQTG<br>GSLRLTCAASGRTSRSYGMGWFRQAPGKEREFVSGISWRGDSTGYADSVKGRFTISRDNAKNTVDLQMNS<br>LKPEDTAIYYCAAAAGSAWYGTLYEYDYWGQGTQVTVSS	250
E2-7D12	VHH-VHH	Dimer	EGFR-RNF128	QVQLQESGGGLVQAGGSLRLSCAASGSISGGKGMGWYRQAPGKEREFVAAIGSGAITYYADSVKGRFTISR<br>DNAKNTVYLQMNSLKPEDTAVYYCAVYTTALDEYPYWGQGTQVTVSSLEVLFQGPQVKLEESGGGSVQTG<br>GSLRLTCAASGRTSRSYGMGWFRQAPGKEREFVSGISWRGDSTGYADSVKGRFTISRDNAKNTVDLQMNS<br>LKPEDTAIYYCAAAAGSAWYGTLYEYDYWGQGTQVTVSS	250
9G8-E1	VHH-VHH	Dimer	EGFR-RNF128	EVQLVESGGGLVQAGGSLRLSCAASGRTFSSYAMGWFRQAPGKEREFVVAINWSSGSTYYADSVKGRFTI<br>SRDNAKNTMYLQMNSLKPEDTAVYYCAAGYQINSGNYNFKDYEYDYWGQGTQVTVSSLEVLFQGPQVQLQ<br>ESGGGLVQAGGSLRLSCAASGNISVQLDMGWYRQAPGKEREFVAAINQGTTTYYADSVKGRFTISRDNAKN<br>TVYLQMNSLKPEDTAVYYCAVYLYDIWNHPYWGQGTQVTVSS	253
9G8-E2	VHH-VHH	Dimer	EGFR-RNF128	EVQLVESGGGLVQAGGSLRLSCAASGRTFSSYAMGWFRQAPGKEREFVVAINWSSGSTYYADSVKGRFTI<br>SRDNAKNTMYLQMNSLKPEDTAVYYCAAGYQINSGNYNFKDYEYDYWGQGTQVTVSSLEVLFQGPQVQLQ<br>ESGGGLVQAGGSLRLSCAASGSISGGKGMGWYRQAPGKEREFVAAIGSGAITYYADSVKGRFTISRDNAKN<br>TVYLQMNSLKPEDTAVYYCAVYTTALDEYPYWGQGTQVTVSS	253
E1-9G8	VHH-VHH	Dimer	EGFR-RNF128	QVQLQESGGGLVQAGGSLRLSCAASGNISVQLDMGWYRQAPGKEREFVAAINQGTTTYYADSVKGRFTISR<br>DNAKNTVYLQMNSLKPEDTAVYYCAVYLYDIWNHPYWGQGTQVTVSSLEVLFQGPEVQLVESGGGLVQAG<br>GSLRLSCAASGRTFSSYAMGWFRQAPGKEREFVVAINWSSGSTYYADSVKGRFTISRDNAKNTMYLQMNS<br>LKPEDTAVYYCAAGYQINSGNYNFKDYEYDYWGQGTQVTVSS	253
E2-9G8	VHH-VHH	Dimer	EGFR-RNF128	QVQLQESGGGLVQAGGSLRLSCAASGSISGGKGMGWYRQAPGKEREFVAAIGSGAITYYADSVKGRFTISR<br>DNAKNTVYLQMNSLKPEDTAVYYCAVYTTALDEYPYWGQGTQVTVSSLEVLFQGPEVQLVESGGGLVQAG<br>GSLRLSCAASGRTFSSYAMGWFRQAPGKEREFVVAINWSSGSTYYADSVKGRFTISRDNAKNTMYLQMNS<br>LKPEDTAVYYCAAGYQINSGNYNFKDYEYDYWGQGTQVTVSS	253
9G8-D1	VHH-VHH	Dimer	EGFR-RNF130	EVQLVESGGGLVQAGGSLRLSCAASGRTFSSYAMGWFRQAPGKEREFVVAINWSSGSTYYADSVKGRFTI<br>SRDNAKNTMYLQMNSLKPEDTAVYYCAAGYQINSGNYNFKDYEYDYWGQGTQVTVSSLEVLFQGPQVQLQ<br>ESGGGLVQAGGSLRLSCAASGTISFIGYMGWYRQAPGKERELVASIASGTSTYYADSVKGRFTISRDNAKNT<br>VYLQMNSLKPEDTAVYYCAATQYIQDVHRYWGQGTQVTVSS	253
D1-9G8	VHH-VHH	Dimer	EGFR-RNF130	QVQLQESGGGLVQAGGSLRLSCAASGTISFIGYMGWYRQAPGKERELVASIASGTSTYYADSVKGRFTISRD<br>NAKNTVYLQMNSLKPEDTAVYYCAATQYIQDVHRYWGQGTQVTVSSLEVLFQGPEVQLVESGGGLVQAGG<br>SLRLSCAASGRTFSSYAMGWFRQAPGKEREFVVAINWSSGSTYYADSVKGRFTISRDNAKNTMYLQMNSLK<br>PEDTAVYYCAAGYQINSGNYNFKDYEYDYWGQGTQVTVSS	253
7D12-D1	VHH-VHH	Dimer	EGFR-RNF130	QVKLEESGGGSVQTGGSLRLTCAASGRTSRSYGMGWFRQAPGKEREFVSGISWRGDSTGYADSVKGRFTI<br>SRDNAKNTVDLQMNSLKPEDTAIYYCAAAAGSAWYGTLYEYDYWGQGTQVTVSSLEVLFQGPQVQLQESG<br>GGLVQAGGSLRLSCAASGTISFIGYMGWYRQAPGKERELVASIASGTSTYYADSVKGRFTISRDNAKNTVYL<br>QMNSLKPEDTAVYYCAATQYIQDVHRYWGQGTQVTVSS	250
D1-7D12	VHH-VHH	Dimer	EGFR-RNF130	QVQLQESGGGLVQAGGSLRLSCAASGTISFIGYMGWYRQAPGKERELVASIASGTSTYYADSVKGRFTISRD<br>NAKNTVYLQMNSLKPEDTAVYYCAATQYIQDVHRYWGQGTQVTVSSLEVLFQGPQVKLEESGGGSVQTGG<br>SLRLTCAASGRTSRSYGMGWFRQAPGKEREFVSGISWRGDSTGYADSVKGRFTISRDNAKNTVDLQMNSL<br>KPEDTAIYYCAAAAGSAWYGTLYEYDYWGQGTQVTVSS	250
7D12-A5	VHH-VHH	Dimer	EGFR-RNF167	QVKLEESGGGSVQTGGSLRLTCAASGRTSRSYGMGWFRQAPGKEREFVSGISWRGDSTGYADSVKGRFTI<br>SRDNAKNTVDLQMNSLKPEDTAIYYCAAAAGSAWYGTLYEYDYWGQGTQVTVSSLEVLFQGPQVQLQESG<br>GGLVQAGGSLRLSCAASGSIFRLWYMGWYRQAPGKEREFVASIGIGATTNYADSVKGRFTISRDNAKNTVYL<br>QMNSLKPEDTAVYYCAVFGWAYSGYHDDFLYWGQGTQVTVSS	254
9G8-A5	VHH-VHH	Dimer	EGFR-RNF167	EVQLVESGGGLVQAGGSLRLSCAASGRTFSSYAMGWFRQAPGKEREFVVAINWSSGSTYYADSVKGRFTI<br>SRDNAKNTMYLQMNSLKPEDTAVYYCAAGYQINSGNYNFKDYEYDYWGQGTQVTVSSLEVLFQGPQVQLQ<br>ESGGGLVQAGGSLRLSCAASGSIFRLWYMGWYRQAPGKEREFVASIGIGATTNYADSVKGRFTISRDNAKN<br>TVYLQMNSLKPEDTAVYYCAVFGWAYSGYHDDFLYWGQGTQVTVSS	257

Table S2

C

NAME	TYPE	FORMAT	DESIGN	AA SEQUENCE	#
DA10-E1	scFv-VHH	Dimer	EPOR-RNF128	EVQLLESGGGLVQPGGSLRLSCAASGFTFSSYAMSWVRQAPGKGLEWVSAISGSGGSTYYADSVKGRFTIS<br>RDNSKNTLYLQMNSLRAEDTAVYYCVKDRVAVAGKGSYYFDSWGRGTTVTVSSGGGGSGGGGSGGGGS<br>QSVLTQPPSVSEAPGQRVTIACSGSSSNIGNNAVSWYQQLPGKAPTLLIYYDNLLPSGVSDRFSGSKSGTSA<br>SLAISGLQSEDEADYYCAAWDDSLNDWVFGGGTKVTVLQVQLQESGGGLVQAGGSLRLSCAASGNISVQL<br>DMGWYRQAPGKEREFVAAINQGTTTYYADSVKGRFTISRDNAKNTVYLQMNSLKPEDTAVYYCAVYLYDIW<br>NHPYWGQGTQVTVSS	367
DA10-A7	scFv-VHH	Dimer	EPOR-RNF43	EVQLLESGGGLVQPGGSLRLSCAASGFTFSSYAMSWVRQAPGKGLEWVSAISGSGGSTYYADSVKGRFTIS<br>RDNSKNTLYLQMNSLRAEDTAVYYCVKDRVAVAGKGSYYFDSWGRGTTVTVSSGGGGSGGGGSGGGGS<br>QSVLTQPPSVSEAPGQRVTIACSGSSSNIGNNAVSWYQQLPGKAPTLLIYYDNLLPSGVSDRFSGSKSGTSA<br>SLAISGLQSEDEADYYCAAWDDSLNDWVFGGGTKVTVL	249
DA10-A10	scFv-VHH	Dimer	EPOR-ZNRF3	EVQLLESGGGLVQPGGSLRLSCAASGFTFSSYAMSWVRQAPGKGLEWVSAISGSGGSTYYADSVKGRFTIS<br>RDNSKNTLYLQMNSLRAEDTAVYYCVKDRVAVAGKGSYYFDSWGRGTTVTVSSGGGGSGGGGSGGGGS<br>QSVLTQPPSVSEAPGQRVTIACSGSSSNIGNNAVSWYQQLPGKAPTLLIYYDNLLPSGVSDRFSGSKSGTSA<br>SLAISGLQSEDEADYYCAAWDDSLNDWVFGGGTKVTVL	249
PD1-E1	VHH-VHH	Dimer	PD1-RNF128	DVQLVESGGGVVQPGGSLRLSCAASGSIASIHAMGWFRQAPGKEREFVAVITWSGGITYYADSVKGRFTISR<br>DNSKNTVYLQMNSLRPEDTALYYCAGDKHQSSWYDYWGQGTLVTVSSLEVLFQGPQVQLQESGGGLVQA<br>GGSLRLSCAASGNISVQLDMGWYRQAPGKEREFVAAINQGTTTYYADSVKGRFTISRDNAKNTVYLQMNSL<br>KPEDTAVYYCAVYLYDIWNHPYWGQGTQVTVSS	245
PD1-E2	VHH-VHH	Dimer	PD1-RNF128	DVQLVESGGGVVQPGGSLRLSCAASGSIASIHAMGWFRQAPGKEREFVAVITWSGGITYYADSVKGRFTISR<br>DNSKNTVYLQMNSLRPEDTALYYCAGDKHQSSWYDYWGQGTLVTVSSLEVLFQGPQVQLQESGGGLVQA<br>GGSLRLSCAASGSISGGKGMGWYRQAPGKEREFVAAIGSGAITYYADSVKGRFTISRDNAKNTVYLQMNSL<br>KPEDTAVYYCAVYTTALDEYPYWGQGTQVTVSS	245
A1-PD1	VHH-VHH	Dimer	PD1-RNF130	QVQLQESGGGLVQAGGSLRLSCAASGYISGYYVMGWYRQAPGKEREFVASISYGASTYYADSVKGRFTISR<br>DNAKNTVYLQMNSLKPEDTAVYYCAVDFDSNYAHTYWGQGTQVTVSSLEVLFQGPDVQLVESGGGVVQPG<br>GSLRLSCAASGSIASIHAMGWFRQAPGKEREFVAVITWSGGITYYADSVKGRFTISRDNSKNTVYLQMNSLR<br>PEDTALYYCAGDKHQSSWYDYWGQGTLVTVSS	245
D1-PD1	VHH-VHH	Dimer	PD1-RNF130	QVQLQESGGGLVQAGGSLRLSCAASGTISFIGYMGWYRQAPGKERELVASIASGTSTYYADSVKGRFTISRD<br>NAKNTVYLQMNSLKPEDTAVYYCAATQYIQDVHRYWGQGTQVTVSSLEVLFQGPDVQLVESGGGVVQPGG<br>SLRLSCAASGSIASIHAMGWFRQAPGKEREFVAVITWSGGITYYADSVKGRFTISRDNSKNTVYLQMNSLRP<br>EDTALYYCAGDKHQSSWYDYWGQGTLVTVSS	245
PD1-A5	VHH-VHH	Dimer	PD1-RNF167	DVQLVESGGGVVQPGGSLRLSCAASGSIASIHAMGWFRQAPGKEREFVAVITWSGGITYYADSVKGRFTISR<br>DNSKNTVYLQMNSLRPEDTALYYCAGDKHQSSWYDYWGQGTLVTVSSLEVLFQGPQVQLQESGGGLVQA<br>GGSLRLSCAASGSIFRLWYMGWYRQAPGKEREFVASIGIGATTNYADSVKGRFTISRDNAKNTVYLQMNSLK<br>PEDTAVYYCAVFGWAYSGYHDDFLYWGQGTQVTVSS	249
A5-PD1	VHH-VHH	Dimer	PD1-RNF167	QVQLQESGGGLVQAGGSLRLSCAASGSIFRLWYMGWYRQAPGKEREFVASIGIGATTNYADSVKGRFTISR<br>DNAKNTVYLQMNSLKPEDTAVYYCAVFGWAYSGYHDDFLYWGQGTQVTVSSLEVLFQGPDVQLVESGGG<br>VVQPGGSLRLSCAASGSIASIHAMGWFRQAPGKEREFVAVITWSGGITYYADSVKGRFTISRDNSKNTVYLQ<br>MNSLRPEDTALYYCAGDKHQSSWYDYWGQGTLVTVSS	249
E2-E2	VHH-VHH	Dimer	RNF128-RNF128	QVQLQESGGGLVQAGGSLRLSCAASGSISGGKGMGWYRQAPGKEREFVAAIGSGAITYYADSVKGRFTISR<br>DNAKNTVYLQMNSLKPEDTAVYYCAVYTTALDEYPYWGQGTQVTVSSGSLEVLFQGPGSQVQLQESGGGL<br>VQAGGSLRLSCAASGSISGGKGMGWYRQAPGKEREFVAAIGSGAITYYADSVKGRFTISRDNAKNTVYLQM<br>NSLKPEDTAVYYCAVYTTALDEYPYWGQGTQVTVSS	248
A1-A1	VHH-VHH	Dimer	RNF130-RNF130	QVQLQESGGGLVQAGGSLRLSCAASGYISGYYVMGWYRQAPGKEREFVASISYGASTYYADSVKGRFTISR<br>DNAKNTVYLQMNSLKPEDTAVYYCAVDFDSNYAHTYWGQGTQVTVSSGSLEVLFQGPGSQVQLQESGGGL<br>VQAGGSLRLSCAASGYISGYYVMGWYRQAPGKEREFVASISYGASTYYADSVKGRFTISRDNAKNTVYLQM<br>NSLKPEDTAVYYCAVDFDSNYAHTYWGQGTQVTVSS	248
A5-A5	VHH-VHH	Dimer	RNF167-RNF167	QVQLQESGGGLVQAGGSLRLSCAASGSIFRLWYMGWYRQAPGKEREFVASIGIGATTNYADSVKGRFTISR<br>DNAKNTVYLQMNSLKPEDTAVYYCAVFGWAYSGYHDDFLYWGQGTQVTVSSGSLEVLFQGPGSQVQLQE<br>SGGGLVQAGGSLRLSCAASGSIFRLWYMGWYRQAPGKEREFVASIGIGATTNYADSVKGRFTISRDNAKNT<br>VYLQMNSLKPEDTAVYYCAVFGWAYSGYHDDFLYWGQGTQVTVSS	256
A7-A7	VHH-VHH	Dimer	RNF43-RNF43	QVQLQESGGGLVQAGGSLRLSCAASGTIFGLSDMGWYRQAPGKEREFVAAIAGGTITYYADSVKGRFTISRD<br>NAKNTVYLQMNSLKPEDTAVYYCAAYKYDERYHSYWGQGTQVTVSSGSLEVLFQGPGSQVQLQESGGGLV<br>QAGGSLRLSCAASGTIFGLSDMGWYRQAPGKEREFVAAIAGGTITYYADSVKGRFTISRDNAKNTVYLQMNS<br>LKPEDTAVYYCAAYKYDERYHSYWGQGTQVTVSS	248
A10-A10	VHH-VHH	Dimer	ZNRF3-ZNRF3	QVQLQESGGGLVQAGGSLRLSCAASGTISYAHIMGWYRQAPGKERELVAGISQGSITNYADSVKGRFTISRD<br>NAKNTVYLQMNSLKPEDTAVYYCAVISYDYIKSVPFRYWGQGTQVTVSSGSLEVLFQGPGSQVQLQESGGG<br>LVQAGGSLRLSCAASGTISYAHIMGWYRQAPGKERELVAGISQGSITNYADSVKGRFTISRDNAKNTVYLQM<br>NSLKPEDTAVYYCAVISYDYIKSVPFRYWGQGTQVTVSS	254
C8-A10	VHH-VHH	Dimer	RNF43-ZNRF3	QVQLQESGGGLVQAGGSLRLSCAASGSIFWKPVMGWYRQAPGKEREFVAAITSGTNTYYADSVKGRFTISR<br>DNAKNTVYLQMNSLKPEDTAVYYCAVDDYDVVEYPYWGQGTQVTVSSGSLEVLFQGPGSQVQLQESGGG<br>LVQAGGSLRLSCAASGTISYAHIMGWYRQAPGKERELVAGISQGSITNYADSVKGRFTISRDNAKNTVYLQM<br>NSLKPEDTAVYYCAVISYDYIKSVPFRYWGQGTQVTVSS	251
C8-E2-A10	VHH-VHH-VHH	Trimer	RNF43-RNF128-ZNRF3	QVQLQESGGGLVQAGGSLRLSCAASGSIFWKPVMGWYRQAPGKEREFVAAITSGTNTYYADSVKGRFTISR<br>DNAKNTVYLQMNSLKPEDTAVYYCAVDDYDVVEYPYWGQGTQVTVSSGSLEVLFQGPGSQVQLQESGGG<br>LVQAGGSLRLSCAASGSISGGKGMGWYRQAPGKEREFVAAIGSGAITYYADSVKGRFTISRDNAKNTVYLQ<br>MNSLKPEDTAVYYCAVYTTALDEYPYWGQGTQVTVSSGSLEVLFQGPGSQVQLQESGGGLVQAGGSLRLS<br>CAASGTISYAHIMGWYRQAPGKERELVAGISQGSITNYADSVKGRFTISRDNAKNTVYLQMNSLKPEDTAVY<br>YCAVISYDYIKSVPFRYWGQGTQVTVSS	381

Table S2

Table S2. REULR Sequences.

(A-C) Table of REULR format, architecture and amino acid sequences.
